# Tetrahydrocarbazoles are a novel class of potent P-type ATPase inhibitors with antifungal activity

**DOI:** 10.1371/journal.pone.0188620

**Published:** 2018-01-02

**Authors:** Maike Bublitz, Lasse Kjellerup, Karen O’Hanlon Cohrt, Sandra Gordon, Anne Louise Mortensen, Johannes D. Clausen, Thomas David Pallin, John Bondo Hansen, Anja Thoe Fuglsang, William Dalby-Brown, Anne-Marie L. Winther

**Affiliations:** 1 Department of Biochemistry, University of Oxford, Oxford, United Kingdom; 2 Pcovery, Copenhagen N, Denmark; 3 Department of Plant and Environmental Sciences, University of Copenhagen, Frederiksberg, Denmark; 4 Charles River Laboratories, Harlow, United Kingdom; Cinvestav-IPN, MEXICO

## Abstract

We have identified a series of tetrahydrocarbazoles as novel P-type ATPase inhibitors. Using a set of rationally designed analogues, we have analyzed their structure-activity relationship using functional assays, crystallographic data and computational modeling. We found that tetrahydrocarbazoles inhibit adenosine triphosphate (ATP) hydrolysis of the fungal H^+^-ATPase, depolarize the fungal plasma membrane and exhibit broad-spectrum antifungal activity. Comparative inhibition studies indicate that many tetrahydrocarbazoles also inhibit the mammalian Ca^2+^-ATPase (SERCA) and Na^+^,K^+^-ATPase with an even higher potency than Pma1. We have located the binding site for this compound class by crystallographic structure determination of a SERCA-tetrahydrocarbazole complex to 3.0 Å resolution, finding that the compound binds to a region above the ion inlet channel of the ATPase. A homology model of the *Candida albicans* H^+^-ATPase based on this crystal structure, indicates that the compounds could bind to the same pocket and identifies pocket extensions that could be exploited for selectivity enhancement. The results of this study will aid further optimization towards selective H^+^-ATPase inhibitors as a new class of antifungal agents.

## Introduction

Invasive fungal infections (IFIs) are a significant threat to human health, especially among immunocompromised, elderly or hospitalized individuals. Despite the availability of a number of treatments, IFIs result in approximately 1.5 million deaths worldwide annually [1]. IFIs are generally associated with high mortality rates, often above 50%, and can approach 90% for some infections. The major IFIs are caused by *Candida*, *Aspergillus* and *Cryptococcus* species [1]. Many of the currently available therapies exhibit poor toxicology profiles (amphotericin B) [2], extensive drug-drug interactions (azoles), and are beginning to suffer from acquired resistance among pathogenic species (azoles and echinocandins) [3,4]. In addition, many antifungals have a limited spectrum of activity and appropriate treatment is often delayed by challenges in diagnosis [5]. Consequently, safer, broad-spectrum antifungal drugs with novel mechanisms of action are urgently required [6].

The fungal H^+^-ATPase Pma1 belongs to a family of membrane-embedded ATPases that pump ions across cellular membranes, a process energized through transient phosphorylation by ATP. Pma1 pumps H^+^ out of the cell, generating a large membrane potential, which drives secondary transporters to import ions and metabolites, such as glucose and amino acids [7]. Pma1 has been shown to be an essential membrane protein through *pma1* gene disruption, RNA interference studies [8] and loss-of-function mutations of Pma1 in yeast [9]. Pma1 is present in all fungi with a high degree of sequence similarity among diverse fungal *pma1* genes (50–96%) but is not present in mammalian cells. A selective Pma1 inhibitor is therefore very likely to have broad-spectrum antifungal activity and no target associated toxicity. Several clinically important therapeutics target other members of the P-type ATPase family. For example, cardiac glycosides target the Na^+^,K^+^-ATPase and proton pump inhibitors (PPIs), such as omeprazole, target the gastric H^+^,K^+^-ATPase [10]. The establishment of the P-Type ATPases as a druggable class of targets suggests that it should be possible to develop Pma1 inhibitors as potent antifungal agents. Notably, Pma1 inhibitors could act from the extracellular side, similar to PPIs, and circumvent the challenges associated with crossing the fungal plasma membrane.

Here we report a compound library screening campaign that identified a series of Pma1 inhibitors that exhibit broad-spectrum antifungal activity. Computer modeling, supported by structural biology indicates that the compounds bind to a groove at the intracellular membrane interface, similar to other P-type ATPase inhibitors that block the ion entry channel.

## Results

### Tetrahydrocarbozole compounds inhibit ATP hydrolysis by P-type ATPases

**Fig 1:**
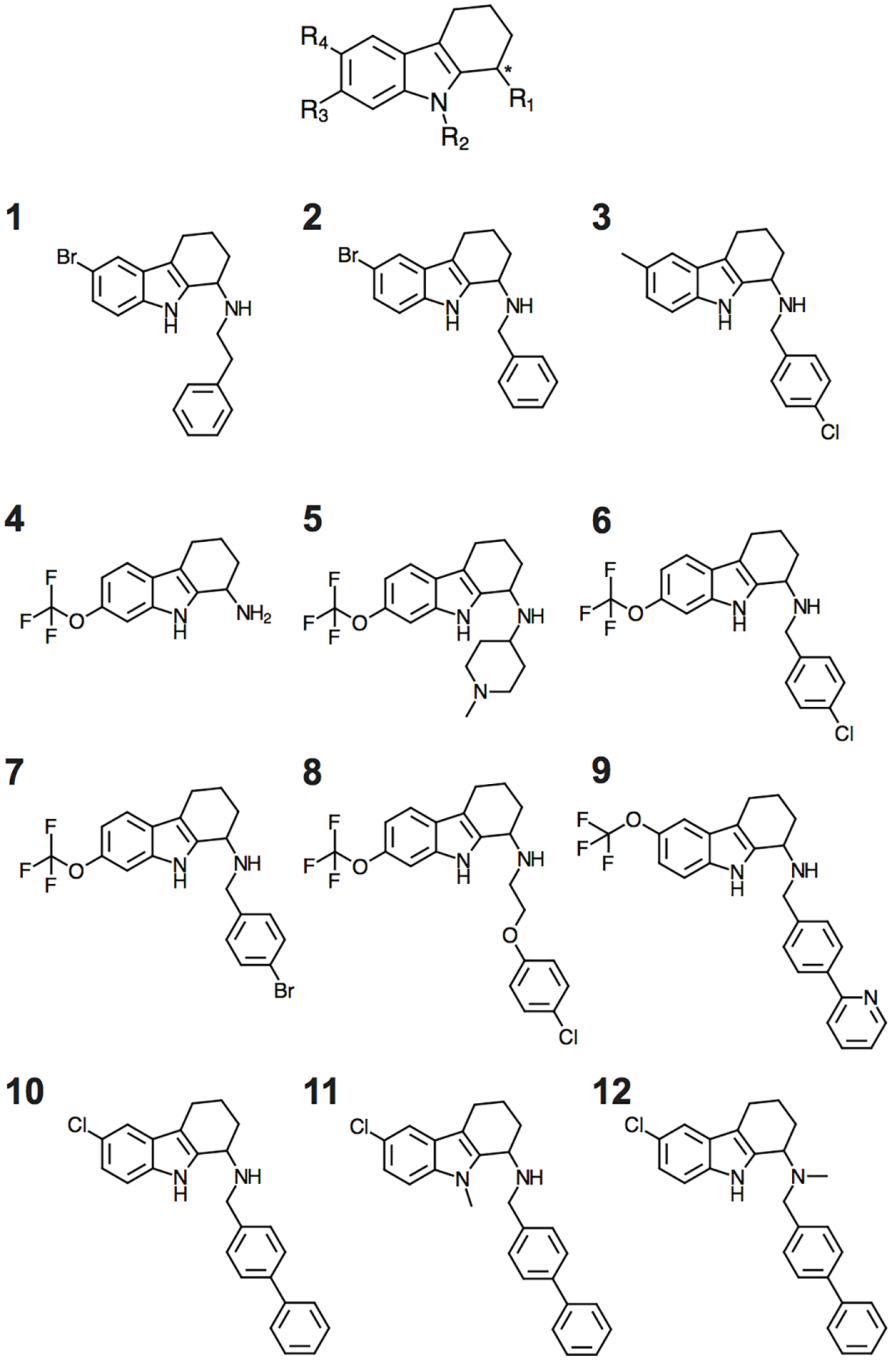
Chemical structure of the tetrahydrocarbazole scaffold (top), three initial hits from the library screening (1–3) and nine rationally designed tetrahydrocarbazole analogues used in this study (4–12). The chiral center is indicated by an asterisk.

20,240 compounds were screened for inhibition of Pma1 ATP hydrolysis at a single concentration (20 μM) to identify initial hits with potency in the low micromolar range. One hundred compounds exhibited >30% Pma1 inhibition, and were selected for Pma1 IC_50_ determination, and then evaluated for antifungal activity against *Saccharomyces cerevisiae* (baker’s yeast) and *Candida albicans* (ATCC 90028). Two-thirds of the Pma1 inhibitors also inhibited fungal growth of *S*. *cerevisiae* and *C*. *albicans* at concentrations below < 100 μM, while the remaining compounds did not inhibit fungal growth. A series of tetrahydrocarbozoles stood out as inhibitors of both Pma1 activity and fungal growth in the low micromolar range (<20 μM). These compounds were repurchased and confirmed as Pma1 inhibitors (compound **1**–**3**, Fig 1, Table 1).

**Table 1:** ATP hydrolysis and growth inhibition by library hit compounds. MIC is defined as 50% growth inhibition, *n* = 3.

	ATP hydrolysis IC_50_ [μM]	Growth inhibition MIC [μM]	
ID	*Saccharomyces cerevisiae* Pma1	*Saccharomyces cerevisiae*	*Candida albicans*
**1**	18.2 ±4.7	10	20
**2**	12.9 ±3.2	12	10
**3**	14.8 ±3.5	12	5

Nine tetrahydrocarbazole compounds (**4**–**12**, Fig 1) were synthesized (see S1 File for details) and the racemic compounds were tested for activity against Pma1 and the related mammalian Ca^2+^-ATPase (SERCA, rabbit) and Na^+^,K^+^-ATPase (pig) (Table 2). Compounds **4** and **5** were weak inhibitors of Pma1, and compounds **11** and **12** exhibited no activity against Pma1. Compounds, **6**–**10** inhibited Pma1 in the low micromolar range (IC_50_ values of 2–9 μM). Compounds **6, 7**, and **8** were more potent in inhibiting SERCA and the Na^+^,K^+^-ATPase than Pma1, and compound **12** was weakly selective for Na^+^,K^+^-ATPase only (IC_50_ = 11.8 μM). Compounds **9** and **10** were potent Pma1 inhibitors (IC_50_ = 5–7 μM) and exhibited antifungal activity in the same concentration range against *Saccharomyces cerevisiae*, *Candida albicans* and *Candida krusei* (Table 3, and see below).

**Table 2:** IC_50_ values of compounds 4–12 against ATP hydrolysis activity of fungal and mammalian P-type ATPases. *n* = 3.

ID	ATP hydrolysis IC_50_ [μM]			
	*Saccharomyces cerevisiae* Pma1	*Candida albicans* Pma1	Mammalian SERCA	Mammalian Na^+^,K^+^-ATPase
**4**	75.0 ±3.2	46.0 ±14.5	1.55 ±0.06	37.2 ±13.6
**5**	85.9 ±10.3	129.7 ±32.2	61.7 ±18.8	131.9 ±35.2
**6**	2.79 ±0.36	3.89 ±1.71	0.09 ±0.03	0.72 ±0.30
**7**	2.43 ±0.39	2.47 ±0.26	0.11 ±0.06	0.38 ±0.12
**8**	3.57 ±1.81	3.77 ±1.04	0.26 ±0.06	1.07 ±0.38
**9**	4.73 ±2.23	5.56 ±1.55	3.27 ±1.89	9.76 ±4.47
**10**	6.69 ±2.06	9.70 ±2.15	5.54 ±3.16	5.50 ±2.74
**11**^a^	>20	>20	>20	>20
**12**^a^	>20	>20	>20	11.8 ±6.8

^a^The compounds were tested in a concentration up to 166 μM, but compound precipitation was observed at concentrations above 20 μM.

**Table 3:** Fungal growth inhibition by compounds 4–12. MIC is defined as 50% growth inhibition, *n* = 3–6. FLC: fluconazole; VRC: voriconazole; AMB: amphotericin B.

	Growth inhibition MIC [μM]					EC_50_ [μM]
ID	*Saccharomyces cerevisiae* ATCC 9763	*Candida albicans* SC5314	*Candida krusei* ATCC 6258	*Candida glabrata* ATCC 90030	*Candida glabrata* Cg003	HepG2 (72 h)
**4**	7.5 ±0.0	100 ±43	66 ±16	23.7 ±0.0	75 ±0.0	3.50 ±1.87
**5**	11.6 ±8.1	150 ±0.0	150 ±0.0	46.5 ±27	>75	4.70 ±3.40
**6**	5.0 ±2.9	7.5 ±0.0	7.5 ±0.0	12.1 ±7.9	>75	16.69 ±3.54
**7**	1.5 ± 0.0	15 ±0.0	4.7 ±0.0	15 ±0.0	>75	14.87 ±0.26
**8**	6.2 ±2.6	7.5 ±0.0	12.9 ±9.4	5.3 ±2.7	>75	13.60 ±1.33
**9**	7.5 ±0.0	7.5 ±0.0	7.5 ±4.8	7.5 ±0.0	23.7 ±0.0	12.45 ±3.68
**10**	7.5 ±0.0	6.8 ±4.6	6.8 ±4.6	21.0 ±6.6	>75	18.64 ±1.12
**11**^a^	>20	>20	>20	>20	>20	>20
**12**^a^	>20	>20	>20	>20	>20	>20
FLC	20.68 ±8.68	0.75 ±0.48	67.17 ±26.98	26.12 ±0.0	>208	>50
VRC	0.27 ±0.19	0.03 ±0.02	1.02 ±0.70	0.61 ±0.28	6.11 ±2.64	>50
AMB	0.02 ±0.01	0.10 ±0.04	0.44 ±0.34	0.10±0.04	0.18±0.08	0.69 ±0.01

^a^The compounds were tested in concentrations up to 75 μM in the fungal growth assay and up to 50 μM against HepG2 cells, but compound precipitation was observed at concentrations above 20 μM.

### Tetrahydrocarbazoles exhibit broad spectrum fungistatic activity

Compounds **6**–**10** exhibited the broadest spectrum of antifungal activity, inhibiting the growth of the yeasts *Saccharomyces cerevisiae*, *Candida albicans*, *Candida krusei* and *Candida glabrata*. The compounds were less potent than voriconazole (VRC) and amphotericin B (AMB) against all fungal isolates tested, but more potent than fluconazole (FLC) on *S*. *cerevisiae*, *C*. *krusei* and *C*. *glabrata* (Table 3). They were also more potent than other, structurally different compound series identified in this and previous library screens, the carbazoles [11] and the pyrido-thieno-pyrimidines [12]. The less potent Pma1 inhibitors **4** and **5** displayed weak antifungal activity. Compounds **11** and **12**, despite their very close structural similarity to **10**, exhibited neither substantial Pma1 inhibition nor antifungal activity (IC_50_ and MIC >20 μM). Finally, one of the synthesized tetrahydrocarbazole compounds, **9**, exhibited antifungal activity against the *C*. *glabrata* strain Cg003, which overexpresses the efflux pumps Cdr1p and Cdr2p associated with multidrug resistance [13].

Compounds were evaluated for mammalian cytotoxicity in a standard human hepatocyte cell proliferation assay (HepG2). All nine tested tetrahydrocarbazoles inhibited HepG2 proliferation (EC_50_: 3–20 μM) more strongly than VRC, but less than AMB (EC_50_: < 1 μM) (Table 3). Compounds **4** and **5** were more potent at inhibiting HepG2 proliferation than fungal growth. Although compounds **6**, **8, 9** and **10** inhibited fungal growth more strongly than HepG2 proliferation, the issue of mammalian cytotoxicity will need to be addressed during further development of the compounds. However, the broad range of EC_50_ values obtained in the proliferation assay, from very cytotoxic to moderate or no toxicity, indicate that the cytotoxicity profile of these compounds can be optimized to increase the selectivity towards fungal cells.

**Fig 2:**
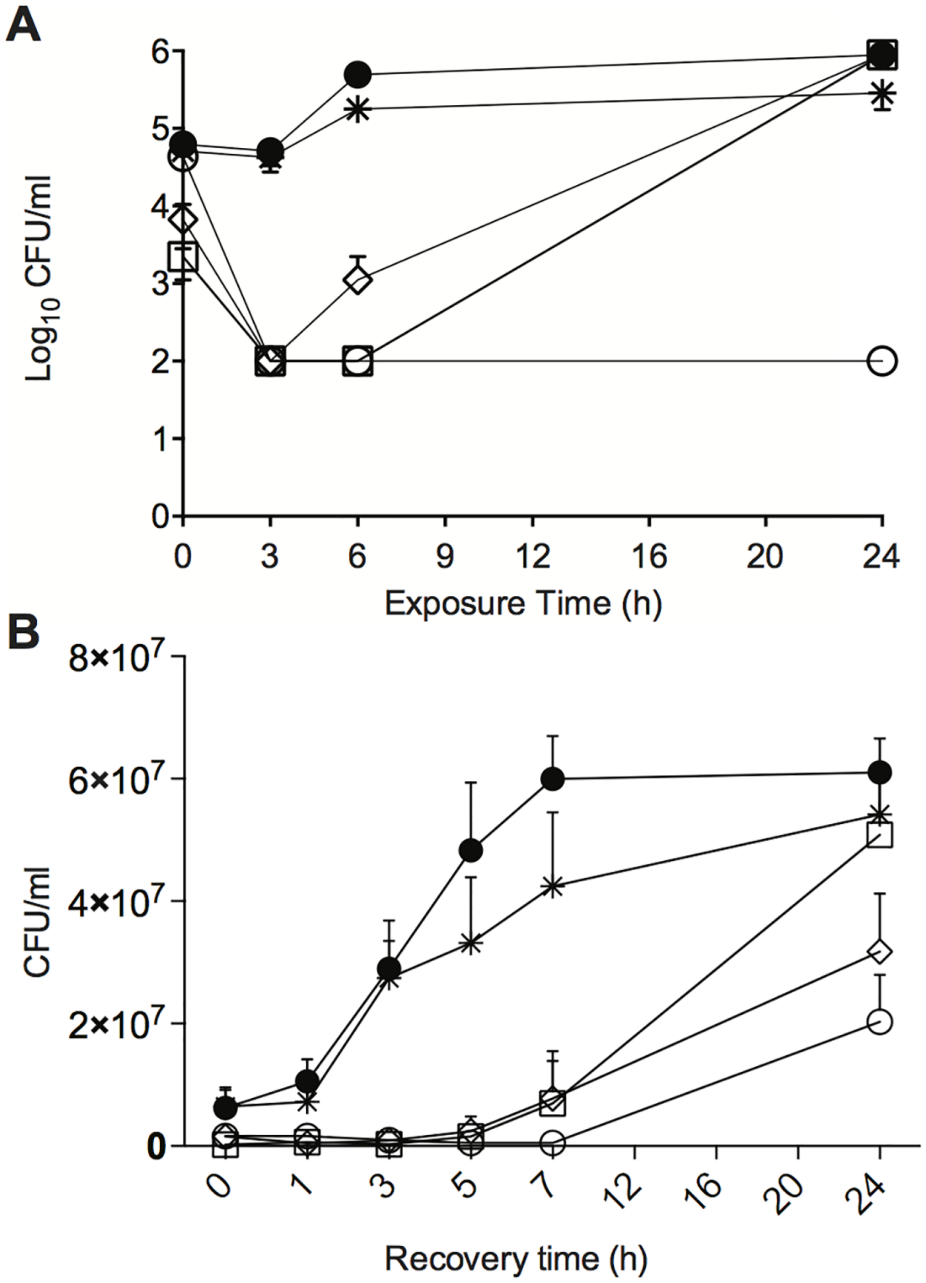
Antifungal activity of tetrahydrocarbazoles against *C*. *albicans*. A) Time-kill analysis and B) Post-antifungal effect of tetrahydrocarbazoles: ● = DMSO-treated control cells, ○ = cells treated with AMB (0.54 μM), * = cells treated with VRC (2.8 μM), □ = cells treated with **6** (20 μM) and ◊ = cells treated with **8** (20 μM). Values in A) and B) are the mean (±SEM) for three independent experiments. Samples withdrawn at the indicated times in A) and B) were evaluated for CFU.

Compounds **6** and **8** were evaluated in time-kill experiments against *Candida albicans*. Here, exposure to **6** or **8** (20 μM) resulted in a rapid reduction in colony-forming units (CFU), compared to dimethylsulfoxide (DMSO) treated controls at T = 0 h (Fig 2A). After 3 h exposure, >99% reduction in CFU was observed for cells treated with **6** or **8**, but after 6 h exposure, these cultures yielded CFU counts similar to DMSO-controls (Fig 2A). The low CFU counts at T = 3 h were similar to observations for AMB, which is fungicidal after 3 h exposure in *C*. *albicans*, and distinct from VRC which exhibits fungistatic activity after both 3 and 24 h of incubation. Thus, the fungal growth data observed for *Candida* cells exposed to **6** and **8** suggests that the tetrahydrocarbazoles both delay growth and displayed fungistatic activity after 24 h of incubation.

Compounds **6** and **8** elicit a long post-antifungal effect (PAFE) after brief exposure in *C*. *albicans* cells (Fig 2B). PAFE is assessed by the ability of fungal cells to grow and divide following compound exposure, and is determined by following the number of CFU/mL in compound-free culture. Fig 2B shows the PAFE after 1 h exposure of *C*. *albicans* to AMB, VRC or compounds **6** or **8**. Exposure to AMB (0.54 μM) significantly delayed the growth of *C*. *albicans* cells, with CFU counts only beginning to rise 7 h after compound removal (PAFE = 7 h). Cells exposed to VRC (2.8 μM) exhibited no significant delay in growth following the same exposure period as compared to untreated cells. Exposure to compounds **6** or **8** (20 μM) led to a 5 h delay in growth as compared to DMSO-treated controls (PAFE = 5 h), a similar effect to that observed with AMB. Once cells that had been exposed to tetrahydrocarbazoles resumed growth they divided rapidly, as evidenced by an exponential increase in CFU counts between 3 and 7 h following compound removal (Fig 2B).

### Tetrahydrocarbazoles induce membrane depolarization without affecting membrane integrity

**Fig 3:**
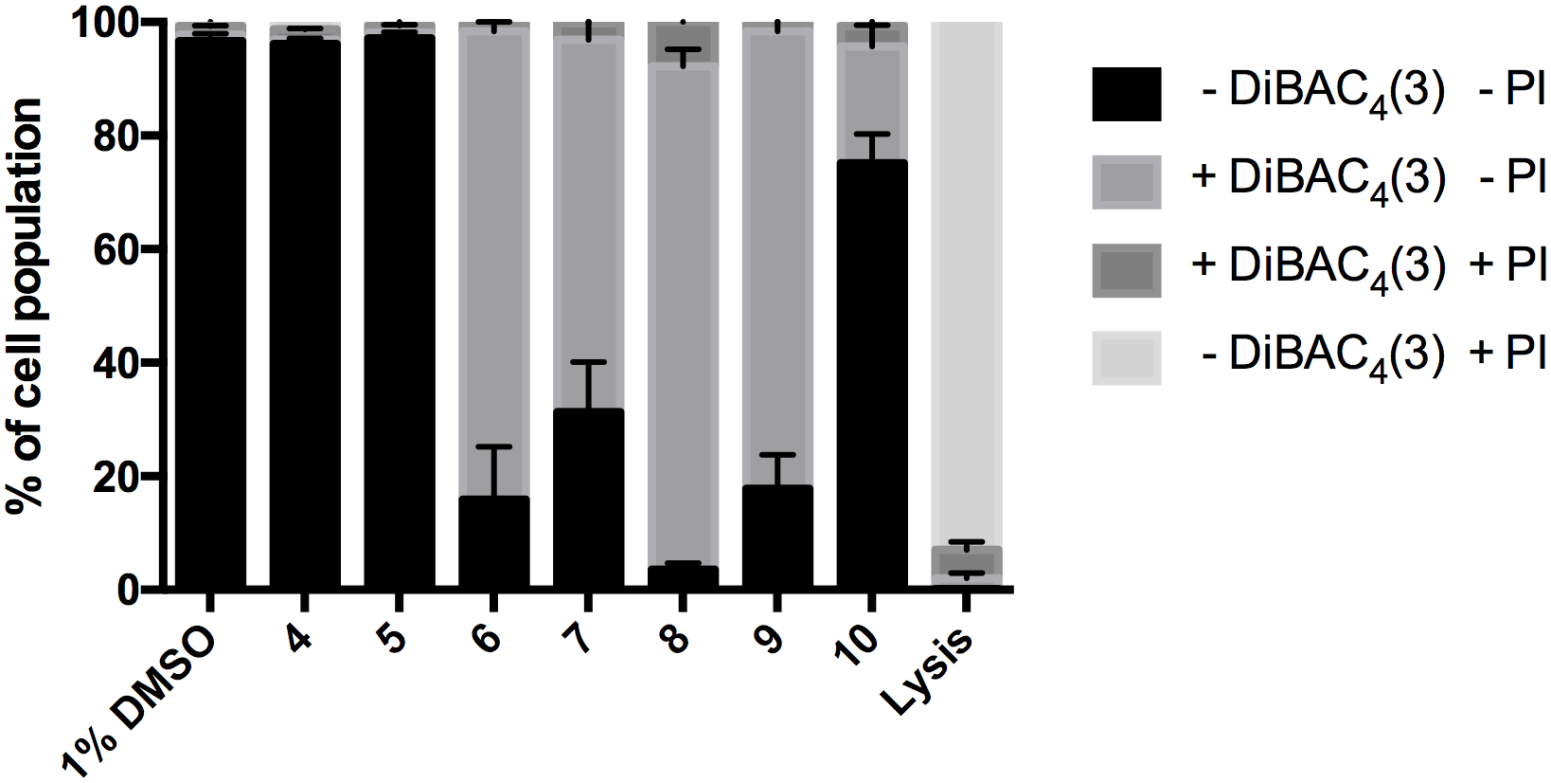
Membrane depolarization effect of tetrahydrocarbazoles. Percentage of fungal cell population measured as PI and DiBAC_4_(3) positive (+) and negative (-) after 30 minutes exposure to tetrahydrocarbazoles (10 μM final concentration). Error bars indicate SEM. Compounds **6**–**10** lead to membrane depolarization (increase in DiBAC_4_(3) signal) with minimal effect on membrane integrity (no increase in the PI signal). *n* = 3 for lysis buffer and *n* = 6 for DMSO control and the compounds.

We assessed the membrane potential and integrity of *S*. *cervevisiae* cells exposed to 10 μM tetrahydrocarbazoles using the fluorescent probes bis-(1,3-dibutylbarbituric acid)trimethine oxonol (DiBAC_4_(3)) and propidium iodide (PI). DiBAC_4_(3) can enter depolarized cells where it binds to intracellular proteins, leading to an enhancement of the fluorescent signal [14]. PI exhibits increased fluorescence upon binding to DNA, and is commonly used as a marker for loss of membrane integrity and the presence of dead cells [15]. No significant differences in the fluorescence signal for DiBAC_4_(3) were observed for *S*. *cerevisiae* cells treated with **4** or **5** as compared to DMSO after 30 minutes exposure (Fig 3). Exposure of *S*. *cerevisiae* to **10** (*S*. *cerevisiae* Pma1 IC_50_ = 7 μM) led to a slight increase in the DiBAC_4_(3) fluorescence intensity, while exposure to the slightly more potent Pma1 inhibitors **6**–**9** (*S*. *cerevisiae* Pma1 IC_50_ = 2–5 μM) led to a significant increase in the fluorescence signal indicative of membrane depolarization induced by these compounds (Fig 3). None of the compounds induced an increase in PI fluorescence, indicating that membrane integrity was not affected. An overview of the results from the membrane potential assays is presented in Fig 3 and Figure A in S1 File.

The observed effects indicate a concurrence of antifungal activity with a loss in membrane potential, which would be consistent with a mechanism involving Pma1 inhibition rather than unspecific membrane damage.

### Crystal structure of an inhibitory P-type ATPase • tetrahydrocarbazole complex

**Fig 4:**
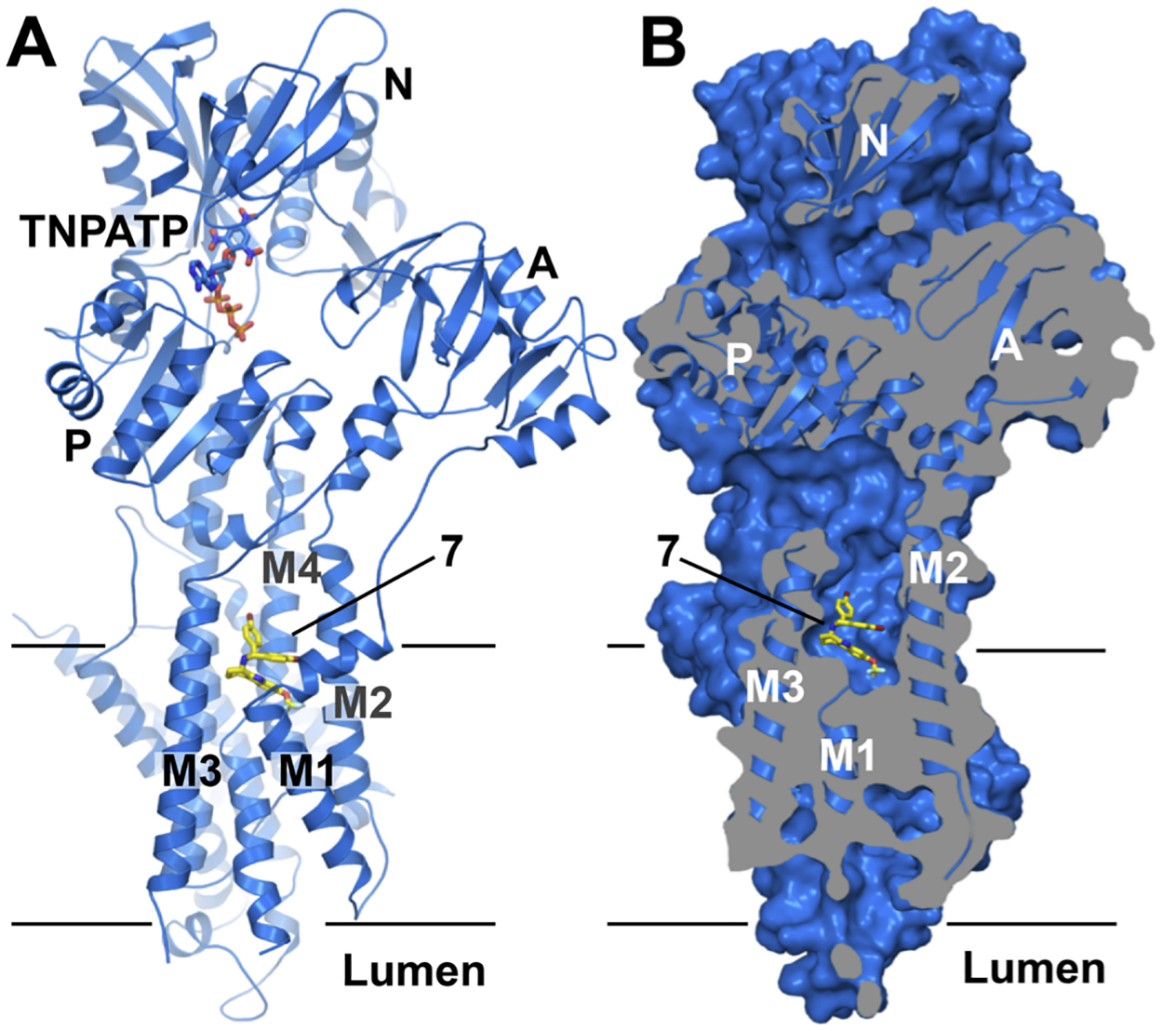
Crystal structure of the SERCA•7•TNPATP complex. A) Cartoon representation of the SERCA•**7**•TNPATP complex. Domains and ligand-binding sites are indicated. Compound **7** (carbon in yellow, oxygen in red, bromine in dark red, fluorine in pale cyan, nitrogen in blue) and TNPATP (carbon in marine, oxygen in red, phosphorous in orange, nitrogen in blue) are shown in stick representation. B) Sliced surface representation revealing the position of the ligand-binding pocket at the cytosolic membrane interface (TNPATP omitted for clarity, colors as in A).

To complement the functional data of the tetrahydrocarbazole compounds with structural information, we collected X-ray diffraction data of an inhibitory complex of compound **7** bound to SERCA (rabbit isoform 1a). This complex was formed in a Ca^2+^-free so-called E2 state and used an ATP analogue, TNPATP, to stabilize the flexible nucleotide-binding region of SERCA, which has been well characterized both functionally and structurally [16–26]. The 3.0 Å crystal structure of the inhibitory complex shows compound **7** bound at the cytosolic membrane interface, in a groove between transmembrane helices M1, M2, M3 and M4 (Fig 4A and 4B).

**Fig 5:**
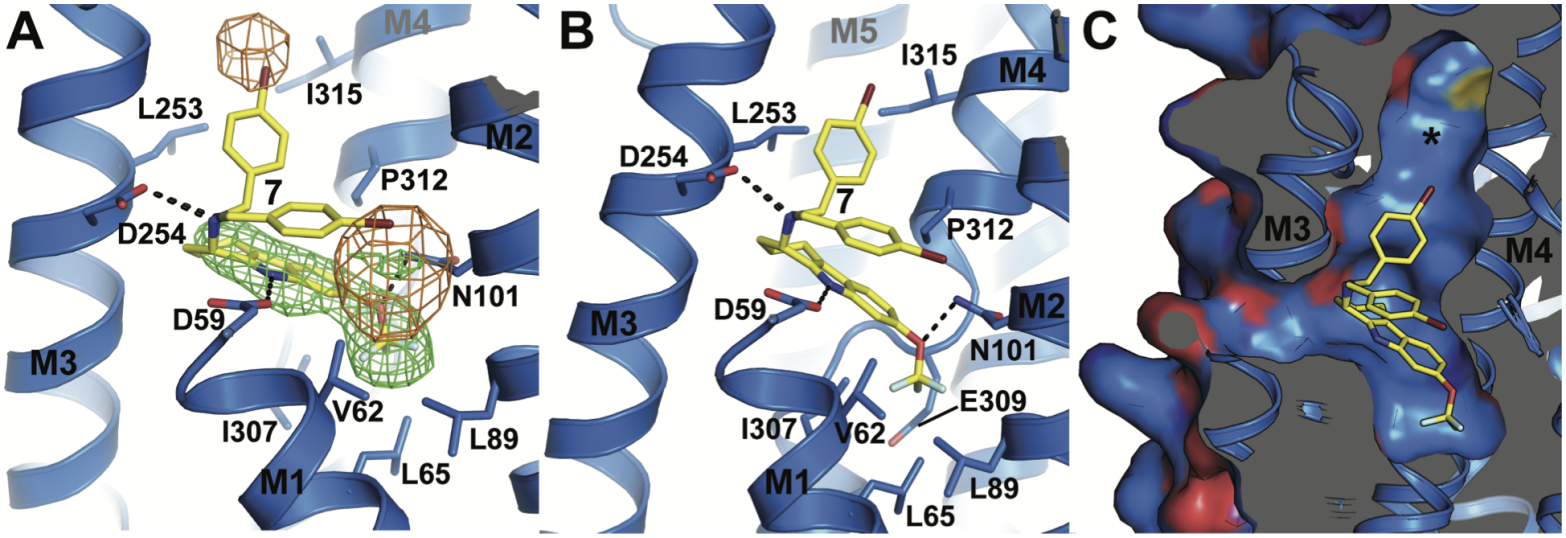
The binding pocket of compound 7. A) Compound **7** with polar and hydrophobic interactions and *m*F_o_-*D*F_c_ difference electron density map before the ligand was modeled (green, contoured at 3 σ) and anomalous difference map (orange, contoured at 4.5 σ, calculated using phases of the final refined model and anomalous difference data from 56–6 Å resolution). B) Slightly rotated view of the ligand binding site, revealing the position of the functionally important residue E309 in SERCA. C) Close-up view of the mostly uncharged surface of the ligand-binding site and its extension towards the P-domain (asterisk). Carbon atoms are colored blue in SERCA and yellow in **7**, oxygen red, nitrogen blue, bromine dark red, and fluorine in pale cyan.

Despite the modest resolution of 3.0 Å, difference electron density maps clearly allow the placement of bound TNPATP at the ATPase active site (Figure B in S1 File), two lipid molecules at the luminal membrane interface (modeled as DOPC, Figure B in S1 File), and of compound **7** which is cradled deep inside the cavity marking the cytoplasmic end of the Ca^2+^ inlet channel of SERCA (Figs 4A, 5A and 5B). Its position superimposes with previously co-crystallized SERCA inhibitors 2,5-di-*t*-butyl-1,4-benzohydroquinone (BHQ) [22] and cyclopiazonic acid (CPA) [25] (Fig 4B and Figure C in S1 File).

The tetrahydrocarbazole core of **7** is clearly visible in an *m*F_o_-*D*F_c_ electron density omit map contoured at 3 σ, but the bromo-phenyl moiety is poorly defined, probably due to an inherent flexibility in the linker connecting it to the tetrahydrocarbazole core. However, an anomalous difference map visualizing the isolated signal from the heavy bromine atom in **7** reveals two distinct peaks (5.7 and 7.3 σ) in close vicinity to the tetrahydrocarbazole core (Fig 5A). A model with two alternative conformations of the bromo-phenyl moiety, with an occupancy of 0.5 each, allowed a straightforward placement of the bromine atoms into both anomalous peaks. One of the two orientations allows for a staggered stacking interaction between the bromo-phenyl group and the tetrahydrocarbazole core. The tetrahydrocarbazole core (modeled with an occupancy of 1) is held in place by three polar contacts to SERCA residues D59 on M1, N101 on M2 and D254 on M3, as well as several hydrophobic interactions involving V62, L65, L98, L253, I307, P312, I315, and the C_β_ and C_γ_ -atoms in the side chain of E309 (Fig 5A and 5B). The pocket extends further towards the inner core of the SERCA protein, bounded by F256 and P308, and also upwards towards the P-domain (Fig 5C, Figure D in S1 File).

Interestingly, the SERCA•7•TNPATP complex protein has crystallized in a previously unreported crystal form, reflecting a novel overall conformation of the ATPase. Structural alignment reveals the most similar known SERCA structures are–as expected–also in the Ca^2+^-free E2 state, stabilized by the inhibitor thapsigargin (Tg) and TNPAMP, TNPADP and TNPATP as nucleotide analogs (PDB ID 3AR5, 3AR6, and 3AR7, all-atom RMSD = 1.90 Å, 1.94 Å and 1.99 Å, respectively). Whereas the transmembrane regions superpose almost identically (all-atom RMSD with 3AR7 = 0.736 Å), the three cytosolic domains (N-, P-, and A-domains) in the SERCA•7•TNPATP structure have undergone a considerable positional shift relative to the transmembrane helices (Figure E in S1 File). The three cytosolic domains adopt positions roughly halfway in-between the Ca^2+^-free E2 and the Ca^2+^-bound E1 state. In particular, the A-domain has undergone a significant movement and tilt (Figure E in S1 File), with the deviation starting at the cytosolic ends of M1 and M2, in the vicinity of the binding site of compound **7** (Figure E in S1 File). It is therefore tempting to speculate that compound **7** induces this new conformation, especially since neither Tg- nor TNPATP-bound SERCA structures display any such substantial conformational changes as compared to their ligand-free counterparts.

### Pma1 homology model and docking of tetrahydrocarbazole compounds

The overall topological organization of all structurally characterized P-type ATPase family members, such as SERCA, the Na^+^,K^+^-ATPase, the plant H^+^-ATPase, the CopA Cu^2+^-ATPase and the Zn^2+^-ATPase [16–18,20,24,27–32], is very similar and it is therefore unlikely that the Pma1 structure deviates significantly from its other family members. We generated homology models of *C*. *albicans* Pma1 and pig Na^+^,K^+^-ATPase based on the SERCA•**7**•TNPATP complex structure, to assess the potential for protein-ligand interactions in the respective binding pockets. In the Pma1 model, the binding pocket is considerably larger than in SERCA, extending deeper into the core of the protein towards transmembrane helix M5, and forming a continuous channel towards the groove between M3, M5, and M7, which is a known binding site of the inhibitor Tg in SERCA (Figure D in S1 File).

**Fig 6:**
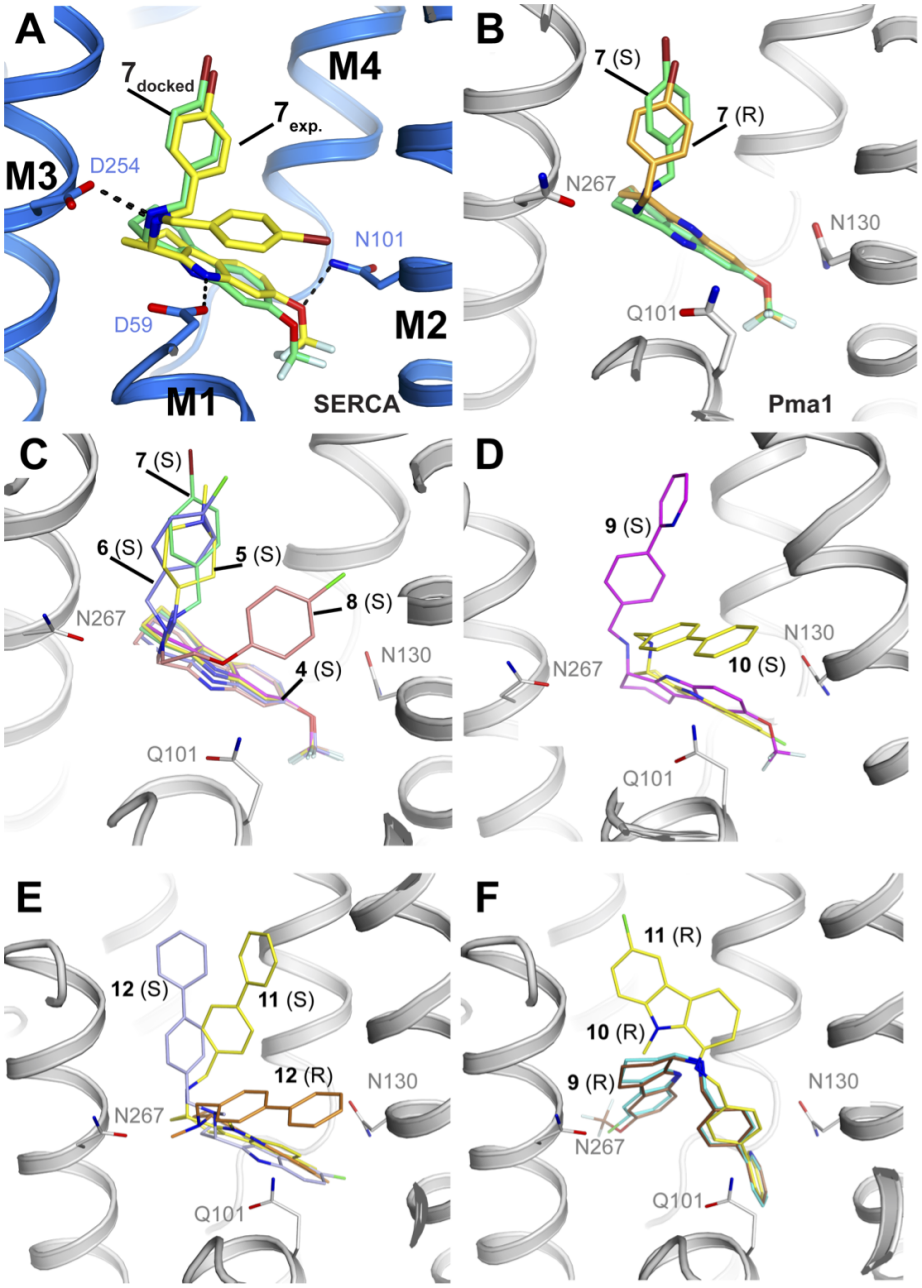
Docking poses of compounds 4–12. Oxygen, nitrogen, bromine, chlorine and fluorine atoms in red, blue, dark red, green, and light blue, respectively. A) Docking (green) and experimental (yellow) binding mode of **7** to SERCA. SERCA is shown as cartoon with carbon in blue, and amino acids D59, D254 and N101 are shown as sticks. Compound **7** is shown as sticks with carbon in yellow. B-F) Docking of **4**–**12** into the *C*. *albicans* Pma1 homology model. The Pma1 model is shown as cartoon with carbon in gray, and amino acids Q101, N130 and N267 are shown as sticks. B) Docking of **7** (S)(green) and **7** (R) (orange) C) Docking of **4** (magenta), **5** (yellow), **6** (blue), **7** (green), **8** (pink) (all S). D) Docking of **9** (S) (magenta), and **10** (S) (yellow). E) Docking of **11** (S)(yellow), **12** (S) (blue), and **12** (R) (orange). F) Docking of **9** (brown), **10** (cyan), and **11** (yellow)(all R).

In an initial control, we attempted to dock both R- and S-isomers of compound **7** back into the SERCA•**7**•TNPATP crystal structure with an empty compound binding site. The docking pose with the second highest affinity score given by the Vina program (-9.2 kcal/mol) for the S-isomer placed the compound almost identical to that modeled in the crystal structure (Fig 6A), whereas the R-isomer did not dock in a similar pose. The highest scoring solution for the S-isomer placed the ligand into the thapsigargin binding groove between M3, M5 and M7, a relatively wide and deep hydrophobic cleft apparently prone to false-positive sampling as we found this site sampled by several of the alternative docking poses throughout. When docked into the Pma1 homology model, however, the highest scoring docking poses of both enantiomers of compound **7** (-9.1 and -9.7 kcal/mol for the R- and S-isomer, respectively) adopt a very similar docking pose to the binding mode observed in the SERCA crystal structure, with key polar interactions to Q101, N267, and N130 (Fig 6B). The highest scoring poses for the other compounds all occupy the same pocket, with relatively similar affinity scores between -8.3 and -9.8 kcal/mol. Generally, compounds with a substituent not larger than a phenyl moiety in position R_1_ (**4**–**8**) favor a docking pose in which the tetrahydrocarbazole core occupies a similar position to that observed in the SERCA•**7**•TNPATP crystal structure. In the enzyme and functional assays the compounds have only been tested as racemic mixtures, but the docking poses of **4–8** are very similar for the S and R-isomers. The R_1_ phenyl groups sample the space between the two conformations observed in the SERCA•**7**•TNPATP complex (Fig 6C). Compounds **9**–**12**, with their larger biphenyl moieties in the R_1_ position, display a larger variety of docking poses. Hence, the S-isomers of compounds **9**–**12** dock with slightly shifted and/or tilted tetrahydrocarbazole cores relative to the configuration of **7**, and with the biphenyl moieties pointing roughly in the same directions as either one of the two conformations observed in **7** (Fig 6D and 6E). With the exception of compound **12**, where both R and S-isomer dock with their tetrahydrocarbazole core in similar positions, the R-isomers of compounds **9**–**11** adopt completely displaced positions within the binding site (Fig 6F).

From our structural and docking data, the poor binding affinity of compounds **11** and **12** for both Pma1 and SERCA can be rationalized by steric clashes in the binding sites. Compound **11** posesses a methyl group at position R_2_ for which there is no space in the tetrahydrocarbazole-binding pocket, resulting in a steric clash with D59 in SERCA, and Q101 in the Pma1 model, and the methyl group on the nitrogen in R_1_ of compound **12** causes a steric clash with D254 (N267 in Pma1): Both compounds display tilted docking poses and less favorable interactions with the protein (Fig 6E).

None of the compounds adopted a docking pose in the Na^+^,K^+^-ATPase homology model that was similar to the observed position of **7** in SERCA (Figure F in S1 File). This is due to the conserved side chain of F100 (Na^+^,K^+^-ATPase pig numbering), which is located at the ‘kink’ of M1 and points into the region where the tetrahydrocarbazole core would be expected to bind. This F100 occupies a roughly equivalent position in all known Na^+^,K^+^-ATPase crystal structures and has previously been pinpointed as the cause for the inactivity of CPA against Na^+^,K^+^-ATPase [25], making the observed inhibitory effect of the tetrahydrocarbazole compounds dependent on an induced fit mechanism or a different binding site.

## Discussion

Our screening approach has yielded a series of tetrahydrocarbazole Pma1 inhibitors with promising antifungal activity. One third of the initial hit compounds exhibited no activity against fungal growth despite being potent inhibitors of ATPase activity *in vitro*, probably due a lack of ability to pass through the fungal cell wall. The physicochemical properties of the fungal cell wall might block passage, compounds might be adsorbed or degraded by cell wall located enzymes. The functional and structural data reveal that the Pma1-inhibitory and antifungal activities of the tetrahydrocarbazoles are strongly dependent on the properties of the R_1_ linker that connects the core with the distal aromats. The most potent Pma1 inhibitors in this study possess an NH-CH_2_-C sequence at R_1_ and only a hydrogen at R_2_.; compounds **6**–**10**. These compounds inhibit the ATP hydrolysis activity of Pma1, display antifungal activity and depolarize the membrane potential of *S*. *cerevisiae* cells. Importantly, the PI signal remained unchanged after 30 minutes of tetrahydrocarbazole exposure, indicating that membrane depolarization was a result of Pma1 inhibition rather than disruption of membrane integrity.

Based on the results from time-kill and PAFE studies and the membrane depolarization and integrity experiments, we propose that **6** and **8** are rapidly taken up into fungal cells leading to inhibition of fungal growth. None of the compounds **(6**–**10**) significantly inhibited the growth of *C*. *glabrata* (Cg003), a fungal strain with up-regulated efflux pump activity (Table 3). This suggests that the compounds might be subject to efflux, although more studies are required to confirm this.

The increased inhibition of Pma1 by **6**–**10** (compared to **4** and **5)** could be due to the increased number of hydrophobic interactions between the binding pocket and the hydrophobic R_1_ carbon chain. Even large biphenyl R_1_ substituents, such as in **9** and **10**, are well tolerated, and these compounds are less potent inhibitors of SERCA and the Na^+^,K^+^-ATPase, as compared to **6**–**8**. Our Pma1 homology model indicates that the larger binding pocket in Pma1, is better placed to accommodate large R_1_ substituents compared to SERCA (Figure D in S1 File). A larger pocket in Pma1 is also consistent with the more permissive docking of both enantiomers of **7**, as compared to SERCA. Compounds **11** and **12** are very close analogs of **10**, but exhibited no inhibitory effect on Pma1 at the concentrations tested. Both compounds differ from **10** by one methyl substitution, at R_2_ in **11** and at the nitrogen of the R_1_ linker in **12**, and their docking attempts gave poses that did not support any favourable hydrogen bonds. In summary, methyl substition at R_2_ is not tolerated. For Pma1 inhibition and antifungal activity the larger aromatic substitution at R_1_ is more favorable for antifungal activity than the shorter substitutions and trifluoromethoxy group together with chloride, bromide, and methyl is well tolerated at the R_4_ position.

Collectively, these data suggest that Pma1 inhibition is the basis for antifungal activity of the tetrahydrocarbazole series presented herein. Of note, the location of the tetrahydrocarbazole binding pocket in SERCA at the cytosolic membrane interface is not directly accessible from the extracytosolic side but requires the compound to pass through the sarcoplasmic reticulum membrane in order to bind. Assuming the tetrahydrocarbazole binding site is located in the corresponding pocket of Pma1, the compounds would have to pass the fungal plasma membrane in order to inhibit Pma1. The exact molecular mechanism of inhibition will need to be established in detail by dedicated kinetic studies, but the SERCA•7•TNPATP structure suggests that tetrahydrocarbazole binding does not interfere with nucleotide binding by SERCA, but instead blocks the entry of ions from the cytosolic side. Compound **7** locks M1, M2, M3 and M4 in a conformation incompatible with calcium binding and transport. The binding pocket extends deep into the core of the protein, reaching through towards the Tg binding groove. In fact, studies have shown how long substituents of Tg analogs can follow an intramembranous course and bridge the two binding pockets [33]. The observed perturbations of M1 and M2, translating into a displacement of the A-domain furthermore induce the formation of a previously unreported tilted conformation of the cytosolic headpiece.

The tetrahydrocarbazoles tested are not specific for Pma1, and also inhibit the mammalian SERCA and the Na^+^,K^+^-ATPase, which may partially explain their inhibitory effects on HepG2 cell proliferation. To advance these compounds in an antifungal drug discovery program an improved selectivity index between mammalian and fungal cells will be required.

Interestingly, the tetrahydrocarbazoles have been identified as potential drug candidates for the treatment of Alzheimer’s disease. **1** and **2** have been identified as compounds which can reduce the enhanced calcium release from the endoplasmic reticulum (ER) in HEK293 cells expressing the familial Alzheimer’s disease (FAD)-linked presenilin 1 mutation [34]. Since tetrahydrocarbazoles inhibit SERCA, it is likely that SERCA inhibition is the reason for normalization of the impaired ER calcium homeostasis by these compounds. Hence, the SERCA•**7**•TNPATP crystal structure could prove useful in optimizing the tetrahydrocarbazole series against SERCA.

Tetrahydrocarbazole derivatives with an amide group at the R_1_ position have been described as potent inhibitors of human papillomaviruses [35–37]. The two most potent tetrahydrocarbazoles with anti-viral activity [36] were synthesized (compound **V1** and **V2**) (Figure G in S1 File). Neither of them inhibited Pma1, nor exhibited antifungal activity against *S*. *cerevisiae* or *C*. *albicans* (Table A in S1 File). A related chemical class of spirotetrahydro-β-carbolines have been identified as antimalarial drug candidates targeting the P-type ATPase PfATP4, a proposed Na^+^ pump regulating cytosolic Na^+^ [38] in the *Plasmodium falciparum* parasite and have undergone a Phase II clinical trial [38–40]. The racemate of the lead compound NITD609 inhibits *Candida albicans* Pma1 and rabbit SERCA in the low micromolar range but is less potent on the pig Na^+^,K^+^-ATPase (Figure H in S1 File and Table B in S1 File). However, the racemate does not have antifungal activity against *C*. *albicans*, *C*. *krusei*, or *C*. *parapsilosis* at concentrations <75 μM. This highlights the importance of the substituent on the tetrahydrocarbazole core for P-type ATPase selectivity and antifungal activity. Interestingly, NITD609 (KAE609) has been suggested to bind to the same hydrophobic cavity in Pma1 [41] as the tetrahydrocarbazoles presented here.

This study laid the structural groundwork for the rational design of selective compounds targeting the fungal H^+^-ATPase. Future work will undertake modifications to the tetrahydrocarbazoles to increase selectivity for Pma1 over mammalian ATPases, to modulate the physicochemical properties to give a favorable *in vivo* pharmacokinetic profile, and eventually demonstrate efficacy in an animal model of fungal infection.

In conclusion, the combined functional and structural insight gained through *in vitro* and cell-based inhibition assays, crystallographic data and computational models has allowed us to identify a novel class of Pma1 inhibitors with broad-spectrum antifungal activity for future optimization towards a drug candidate.

## Materials and methods

Compounds **4**–**12, V1, V2** and racemic of NITD609 were synthesized at Aurigene Discovery Technologies Ltd, India. Fluconazole (FLC) was purchased from Saveen & Werner AB (Sweden). Voriconazole (VRC) and amphotericin B (AMB) were purchased from Sigma-Aldrich (Saint Louis, MO).

### Compound library screening

The compound libraries were purchased from Enamine Ltd, Ukraine and Chembridge Corp, USA. Library screening was performed in 96 well plates format and each compound was screened in the ATP hydrolysis assay at a final concentration of 20 μM. DMSO was used to determine the maximal ATPase activity and a blank with ATP in absence of Pma1 protein was used to determine the level of spontaneous ATP hydrolysis and used to determine background level. The percentage of compound inhibition was evaluated by subtracting the blank from each data point and normalizing to maximal ATP hydrolysis activity. Compounds that exhibited >30% Pma1 inhibition at 20 μM concentration were selected for further evaluation. Two-fold dilution plates of the compounds were used to determine Pma1 IC_50_ and evaluated for fungal growth inhibition against *Saccharomyces cerevisiae* (baker’s yeast) and *Candida albicans* (ATCC 90028) in a concentration range of 100 μM to 1.5 μM. Pma1 inhibitory hit compounds (IC_50_ <20 μM) with antifungal activity on both *S*. *cerevisiae* and *C*. *albicans* (MIC <100 μM) were re-purchased and re-evaluated to confirm the potency and identity of the compound.

### Preparation of fungal membrane fractions

Plasma membranes containing Pma1 were isolated from *Saccharomyces cerevisiae* RS72 cells containing the full-length cDNA of the *S*. *cerevisiae* plasma membrane H^+^-ATPase isoform *PMA1* under control of the *PMA1* promoter as described [42].

*Candida albicans* microsomes were prepared from 100 mL overnight culture in YPD (10 g/L yeast extract (BD, Sparks, MD), 20 g/L Bacto peptone (BD, Sparks, MD), 20 g/L D-(+)-glucose), transferred to 1 L YPD and grown at RT, 150 rpm, for 7 h. Cells were harvested and washed in deionized water followed by centrifugation at 3360 x *g*. The cells were glucose-activated by the addition of 10% (w/v) D-(+)-glucose for 10 minutes while shaking. This suspension was then centrifuged at 3,360 x *g* for 3 minutes and 60–80 g of cells (wet weight) were resuspended in homogenization buffer (50 g/L glucose, 28.3% (v/v) glycerol, 100 mM Tris-HCl pH 7.25, 10 mM EDTA pH 8.0, 50 mM KCl, 1 mM DTT, 200 μM PMSF, 2 μg/ml Pepstatin A), and disrupted with 165 g glassbeads (500 μm diameter) in a BeadBeater (Biospec Product, Bartlesville, OK). The disrupted cells were centrifuged at 4°C for 5 and 15 minutes at 1,400 x *g* and 12,000 x *g*, respectively. The supernatant was collected and centrifuged at 251,000 x *g* for 1 h, and the membrane fraction was collected and homogenized in GTEK_20_ buffer (20% (v/v) glycerol, 10 mM Tris-HCl pH 7.25, 25 mM KCl, 0.5 mM EDTA pH 8.0, 1 mM DTT, 0.2 mM PMSF, 2 μg/mL Pepstatin A). Further plasma membrane purification with a sucrose gradient did not improve purity, hence *C*. *albicans* microsome preparations were used in the ATP hydrolysis assay.

All membrane batches were validated by determining ATPase activity, pH optimum and orthovanadate sensitivity. Sarco/endoplasmatic reticulum (SR) Ca^2+^ ATPase was provided in SR membranes purified by extraction with a low concentration of deoxycholate (DOC) and was kindly provided by Claus E. Olesen and Jesper V. Møller, Aarhus University and prepared as described in [43]. The pig kidney Na^+^,K^+^-ATPase purification included a mild SDS treatment of isolated microsomes followed by a washing step and was kindly provided by Natalya Fedosova, Aarhus University and prepared as described in [44].

### ATP hydrolysis inhibition

ATPase activity was determined by measuring the amount of liberated phosphate from ATP hydrolysis. The ATPase assay was performed in 96 well plates in a final reaction volume of 60 μL. 0.1–0.2 μg/well of DOC extracted SERCA membrane or the Na^+^,K^+^-ATPase was used, while 1–2.5 μg/well was used of the Pma1 membrane preparation. Reactions, including protein membrane preparation and various concentrations of exogenously added compounds, were conducted in the following buffers; Pma1 buffer: 17.5 mM MOPS-NaOH pH 7, 7 mM MgSO_4_, 44 mM KNO_3_ (vacuolar ATPase inhibitor), 22 mM NaN_3_ (mitochondrial ATPase inhibitor), 0.22 mM Na_2_MoO_4_ (acid phosphatase inhibitor); SERCA buffer: 9 mM MOPS-NaOH pH 7, 2.7 mM MgCl_2_, 0.1 mM CaCl_2_ and 72 mM KCl. Na^+^,K^+^-ATPase buffer: 30 mM MOPS-NaOH pH 7, 40 mM NaCl, 4 mM MgCl_2_ and 20 mM KCl. Reactions were initiated by the addition of Na-ATP to a final concentration of 2.5 mM (Pma1 and Na^+^,K^+^-ATPase) or 5 mM (SERCA), followed by 30 minutes incubation at 30°C. The amount of liberated phosphate was determined colorimetrically after addition of STOP solution (mixture of L-ascorbic acid, ammonium heptamolybdate tetrahydrate, and HCl to give final concentrations of 65 mM, 2.2 mM, and 189 mM, respectively) with 5 minutes incubation at room temperature (RT) followed by addition of arsenite solution (mixture of NaAsO_2_, sodium citrate dihydrate, and acetic acid to give final concentrations of 3.1 mM, 28 mM, and 141 mM, respectively). Absorption was measured at 860 nm after additional 30 minutes incubation at RT.

### Fungal growth inhibition

Fungal isolates were purchased from American Type Culture Collection, except for *C*. *glabrata* strain Cg003, which was kindly provided by Julius Subik, Comenius University in Bratislava, Slovak Republic. Cg003 is a clinical isolate, characterized to be resistant to fluconazole and itraconazole due to overexpression of the multidrug resistance efflux pumps Cdr1p and Cdr2p [13]. *Saccharomyces cerevisiae* (ATCC 9763), *Candida albicans* (ATCC 90028), *Candida albicans* (SC5314), *Candida glabrata* (ATCC 90030), *Candida glabrata* (Cg003) and *Candida krusei* (ATCC 6258) were grown in Sabouraud broth (40 g/L D-glucose, 10 g/L peptone, pH 5.6) or YPD (10 g/L yeast extract, 20 g/L Bacto-peptone, 20 g/L glucose) medium to mid log phase, aliquoted into 20% (v/v) glycerol and cryopreserved as stocks at -80°C. For each growth assay, cells from these stocks were diluted in sterile water to OD_600_ = 0.025, which corresponds to a final starting inoculum of 0.5–2.5 x 10^5^ CFU/mL. Growth assays were carried out by pipetting 3 μL compound dissolved in DMSO, 100 μL cell suspension and 97 μL 2 x RPMI media (20.8 g/L RPMI-1640 (Sigma-Aldrich catalog number R6504) 330 mM MOPS, 36 g/L glucose) into a microtiter plate and incubating for 24 h at 34°C, followed by OD measurement at 490 nm. The minimum inhibitory concentration (MIC) was defined as 50% growth inhibition of the microorganism as this measurement best represented the antifungal activity of the compounds. Standard errors were typically below 5%.

### Time-kill assay

*C*. *albicans* (SC5314) cells (1 x 10^5^ CFU/mL) were incubated in 10 mL RPMI-1640 media at 30°C with gentle agitation (150 rpm) in the presence of amphotericin B (0.5 μM), voriconazole (2.8 μM) or tetrahydrocarbazole compound (20 μM). At the indicated time points (0, 3, 6, and 24 h), a 100 μL aliquot was removed, serially diluted (10 fold) in saline (0.9% NaCl), and 30 μL aliquots were spread on YPD agar. CFU were counted after incubation at 30°C for 48 h [45]. *C*. *albicans* cells treated with DMSO (1%, v/v) served as a control. 50 CFU/mL was selected as the lower limit of quantitation.

### Post-antifungal effect determination

The post-antifungal effect (PAFE) of antifungal drugs on *C*. *albicans* was determined as described by others [46], with modifications. Briefly, 2 × 10^6^ CFU/mL *C*. *albicans* cells were incubated in 1 mL RPMI-1640 media for 1 h at 30°C with gentle agitation in the presence of amphotericin B (0.54 μM), voriconazole (2.8 μM) or tetrahydrocarbazole compound (20 μM). Cells were subsequently washed in RPMI media 3 times by centrifugation (3 minutes, 5,000 x g) and finally re-suspended in 1 mL fresh RPMI medium and incubated at 30°C for 24 h. At various time intervals, a 100 μL aliquot was removed, serially diluted (10-fold) in saline (0.9% NaCl), and 30 μL aliquots were spread on YPD agar. The agar plates were incubated at 30°C for 48 h and the number of CFU/mL of culture was determined. *C*. *albicans* cells treated with DMSO (1%, v/v) served as a control. The number of CFU/mL obtained at specified time intervals was plotted against time (h) following compound removal. An increase in the CFU/mL of culture signifies growth and multiplication of yeast cells. The duration required for the antifungal drug-treated cells to recover from the inhibitory effect of the drug as indicated by an increase in CFU/mL of culture is defined as the PAFE.

### Membrane potential and integrity study

*S*. *cerevisiae* cells were grown to mid-log phase, pelleted at 2,000 x *g* for 2 minutes and washed twice in buffer A (100 mM MOPS and 1 mM KCl adjusted with Trizma base to pH 7.0). Cells were then resuspended in buffer A to an OD_600_ of 0.2. For each well 50 μL of buffer A with 3.3 μg/mL propidium iodide (PI) (Thermo Fisher Scientific, catalog number P1304MP) and 1 μg/mL DiBAC_4_(3) (Sigma-Aldrich, catalog number D8189)) was mixed with 50 μL *S*. *cerevisiae* cell suspension and transferred to a 96 well plate containing 1 μL of inhibitor (in DMSO). A lysis control was performed with Reagent Y100 (ChemoMetec). Five thousand cells were measured per experiment with an exposure time of 1000 ms using a NucleoCounter NC-3000 (ChemoMetec). Dark field was used as the masking channel to select the yeast cells, and the DiBAC_4_(3) (ex: 530 nm, em 675/75 nm) and PI (ex 630 nm, em 740/60 nm) channels were used to measure the membrane potential and membrane integrity, respectively. Only cells with a pixel size of 10–40 were included in the analysis, to avoid analysis of non-cell artifacts.

### Hep G2 viability assay

In each well of a 96 well tissue culture plate (Greiner, catalog number GR-655180) 10,000 human hepatocyte HepG2 cells (Sigma-Aldrich, catalog number 85011430) were plated in 200 μL growth media (EMEM (Sigma-Aldrich, catalog number M2279), 2 mM L-Glutamine (Biological Industries, catalog number 03-020-1B), 1% non-essential amino acids (Biosera, catalog number XC-E1154/100), 10% fetal bovine serum (Biological Industries, catalog number BI-04-007-1A)), and incubated overnight at 37°C and 5% CO_2_. The following day, fresh growth media plus 2 μL compound in DMSO was added. The plate was incubated for a further 72 h at 37°C and 5% CO_2_. The media was replaced with 100 μL freshly prepared 0.5 mg/mL XTT sodium salt solution (Sigma-Aldrich, catalog number X4251) in RPMI-1640 (Sigma-Aldrich, catalog number R7509) with 3.83 μg/mL phenazine methosulfate (Sigma-Aldrich, catalog number P9625) and incubated 2–3 h at 37°C and 5% CO_2_. The color reaction was measured on a Victor X5 plate reader (Perkin-Elmer) at OD_450_, and the half maximal effective concentration (EC_50_) was calculated indicating the potency of tetrahydrocarbazole derivatives to inhibit HepG2 cell growth. Tamoxifen (Sigma-Aldrich, catalog number 85256) was used as a positive control compound while DMSO served as a negative control.

### Crystal structure determination

Rabbit sarcoplasmatic reticulum membranes containing SERCA were prepared as in [43]. SERCA membranes (4.3 mg) were resuspended and gently homogenized in 100 mM MOPS-Tris pH 6.8, 80 mM KCl, 3 mM MgCl_2_, 4 mM EGTA and 20% (v/v) glycerol. Compound **7** (0.5 mM final concentration) was added to the SERCA membranes and incubated overnight at 4°C. The following day, the protein was solubilized at a detergent/protein (w/w) ratio of 1.5:1 with C_12_E_8_ followed by 10 minutes incubation before centrifugation (TLA-55 rotor, 50,000 rpm, 20 minutes, 4°C). The concentration of solubilized protein was usually 10–12 mg/mL, to which 0.5 mM TNPATP was added prior to crystallization. Co-crystallization of SERCA with compound **7** was carried out using hanging drop equilibration at 23°C with protein/buffer/detergent in a 1:1:0.25 ratio. The best diffracting crystals were obtained by the addition of 0.1% (w/v) of n-Octyl-β-D-galactopyranoside and buffer consisting of 7.5% glycerol, 21% PEG6000, 100 mM NaOAc pH 7.2, 6% MPD. The crystals were cryoprotected by a 10 second transfer to a cryobuffer consisting of a 1:1 mixture of 25% glycerol and the crystallization buffer. Data was collected at beam line PXIII at the Swiss Light Source (SLS) in Villigen, Switzerland. The data were collected at 100 K and a wavelength of 0.9 Å, and processed using XDS and XSCALE [47]. The structure was determined using individual domains of SERCA (pdb: 2AGV, all ligands removed) and several rounds of molecular replacement using PHASER in PHENIX with identification of the position of the cytoplasmic domains, followed by the transmembrane helices. PHENIX was used for refinement [48], COOT for model building [49] and MOLPROBITY for model validation [50]. Data collection information and refinement statistics are presented in Table C in S1 File. Several structural reports on SERCA and the Na^+^,K^+^-ATPase were included in the model analysis [16–25,28,51]. The final model of SERCA bound to compound **7** deviates significantly from 2AGV (overall rmsd 2.013 Å). 2AGV is very similar to the structure 3AR7, which we compare with SERCA•7•TNPATP in Figure E in S1 File.

### Homology modeling and molecular docking

For the generation of homology models, we used the software programs Schrodinger and the module Prime. The sequence alignment between *Candida albicans* Pma1 and rabbit SERCA (~20% sequence identity) was manually validated and modified using a structure-based alignment of SERCA with the plasma H^+^-ATPase from the plant *Arabidopsis thaliana* AHA2 [32] and a sequence alignment of AHA2 with *C*. *albicans* PMA1 (~40% homology). The final optimized alignment is illustrated in Figure I in S1 File. For building the homology model using Prime, conserved regions were copied, non-conserved side chains replaced and optimized, insertions built and deletions closed. Short loops were refined using the standard sampling protocol, whereas the five longest loops were refined using an extended loop protocol. Molecular dockings were carried out with AutoDock/Vina as follows. The SERCA structure was prepared with AutoDockTools [52] by adding hydrogen atoms and defining a cubic sampling grid box with a side length of 48 Å, centered approximately in the center of the cytosolic boundary of the transmembrane domain, encompassing all transmembrane helix regions above the ion binding sites, the lower third of the P-domain and reaching up to the boundary of the A domain. (Figure J in S1 File). The large size of the search space was chosen in order to prevent a bias towards the experimentally determined binding site. The ligand molecules were built and energy-minimized in MAESTRO (Maestro, Schrödinger, LLC, New York, NY, 2017) and prepared for docking in AutoDockTools, making all relevant bonds rotatable. Nine docking poses were calculated with Vina (using default parameters) for each compound enantiomer on the SERCA crystal structure, and the Pma1 and NKA models. All docking poses depicted in Fig 6 are the top scoring poses according to the Autodock/Vina affinity score (given in kcal/mole).

## Supporting information

S1 File **Figure A: Membrane potential and integrity assay**. *S*. *cerevisiae* cells incubated for 30 min with A: 1% DMSO, B-H: 10 μM **4–10**, I: Lysis buffer. The 4 quadrants define cells as DiBAC_4_(3) positive (+, top quadrants) or negative (-, bottom quadrants) and PI positive (+, right quadrants) and negative (-, left quadrants). **Figure B: Difference electron densities for bound TNPATP and lipids**. A) Bound TNPATP between the N- and P-domain (domain borders indicated by dashed lines). This binding mode is almost identical to previously reports (pdb: 3AR7) (1). B) Residual electron density indicated the presence of two lipid molecules at the luminal membrane interface. In accordance to the expected abundance in a sample derived from SR membrane, two DOPC molecules were modeled. The green mesh is an *m*Fo-*D*Fc electron density map calculated before the ligands were added to the model, and contoured at 3.0 σ and 2.8 σ for TNPATP and DOPC, respectively. **Figure C: Compound 7 binds in the same pocket as BHQ and CPA in SERCA**. Structural alignment of SERCA•7•TNPATP (pdb:5NCQ, blue) with A) SERCA•BHQ (pdb:2AGV, salmon) and B) SERCA•CPA (pdb:3FGO, cyan) (2, 3). The compound is cradled between M1, M2, M3 and M4 (behind the viewpoint) inside the cavity marking the cytoplasmic end of the Ca^2+^ inlet channel of SERCA. The view is from the inside of the cavity, for clarity. C) Chemical structures of BHQ and CPA. **Figure D: Binding pocket of 7 in the SERCA crystal structure and in the Pma1 homology model**. A) Binding pocket of **7** in SERCA. B) The binding pocket of **7** in the Pma1 homology model is larger as compared to the one in the SERCA crystal structure, and it extends deeper into the core of the protein towards transmembrane helix M5 (indicated with a white arrow). **Figure E: SERCA**•**7** •**TNPATP crystal structure (pdb: 5NCQ, blue) superposed with the crystal structure of SERCA**•**Tg** •**TNPATP crystal structure (pdb: 3AR7, orange)**. A) Overall view of the two crystal structures superposed on transmembrane helices 7–10. All three cytosolic domains have undergone a positional shift. B) Top view on the N- and A-domains, superposed on the N-domain, illustrating the considerable displacement of the A domain (labeled A* in the SERCA•7•TNPATP structure), including the functionally important so-called TGES-loop (residues 181–184, red and orange spheres). C) Displacement of transmembrane helices M1 and M2 near the binding site of compound **7**. These displacements translate into a substantial backward movement of the A- domain and smaller displacements of the P- and N-domains as compared to all other known SERCA structures. **Figure F: Docking of THCs into the Na**^**+**^,**K**^**+**^**-ATPase homology model**. Structural overlay of compound **7** (yellow) within the SERCA crystal structure with the docking results of compound **4**–**12** docked into the Na^+^,K^+^-ATPase homology model. None of the compounds gave a docking position similar as to the observed position of **7** in SERCA, due to a steric clash with Phe100, a residue previously discussed to be responsible for the resistance of the Na^+^,K^+^-ATPase to CPA (Laursen, Bublitz et al, 2009)(3). **Figure G: Compound V1 and V2 identified as viral inhibitors**. Compound 25 and 26 in Gudmundsson et al., 2009 (4). **Table A: ATP hydrolysis and fungal growth effect of V1 and V2. Figure H: The racemic compound of NITD609**. Synthesis described in Dandapani et al., 2012 (5). **Table B: ATP hydrolysis and fungal growth effect of the racemic of NITD609. Table C**. Data collection and refinement statistics. **Figure I: Sequence alignment of *Candida albicans* Pma1 and rabbit SERCA used as basis for homology modeling**. Residues with polar interactions to compound **7** and their equivalents in the Pma1 homology model are marked with red asterisks, other residues surrounding the binding site are marked with black asterisks. Note that Q101 in the Pma1 model is at an almost equivalent position as SERCA D59. **Figure J: Graphical representation of the grid box used as sampling space for docking with AutoDock/Vina**.
(PDF)
