# Supplementary material for: Tetrahydrocarbazoles are a novel class of potent P-type ATPase inhibitors with antifungal activity
Source: PLoS One. 2018 Jan 2;13(1):e0188620. doi: 10.1371/journal.pone.0188620 (PMC5749684; doi:10.1371/journal.pone.0188620)
Supplement: S1 File — Figure A: Membrane potential and integrity assay. S. cerevisiae cells incubated for 30 min with A: 1% DMSO, B-H: 10 μM 4–10, I: Lysis buffer. The 4 quadrants define cells as DiBAC4(3) positive (+, top quadrants) or negative (-, bottom quadrants) and PI positive (+, right quadrants) and negative (-, left quadrants). Figure B: Difference electron densities for bound TNPATP and lipids. A) Bound TNPATP between the N- and P-domain (domain borders indicated by dashed lines). This binding mode is almost identical to previously reports (pdb: 3AR7) (1). B) Residual electron density indicated the presence of two lipid molecules at the luminal membrane interface. In accordance to the expected abundance in a sample derived from SR membrane, two DOPC molecules were modeled. The green mesh is an mFo-DFc electron density map calculated before the ligands were added to the model, and contoured at 3.0 σ and 2.8 σ for TNPATP and DOPC, respectively. Figure C: Compound 7 binds in the same pocket as BHQ and CPA in SERCA. Structural alignment of SERCA•7•TNPATP (pdb:5NCQ, blue) with A) SERCA•BHQ (pdb:2AGV, salmon) and B) SERCA•CPA (pdb:3FGO, cyan) (2, 3). The compound is cradled between M1, M2, M3 and M4 (behind the viewpoint) inside the cavity marking the cytoplasmic end of the Ca2+ inlet channel of SERCA. The view is from the inside of the cavity, for clarity. C) Chemical structures of BHQ and CPA. Figure D: Binding pocket of 7 in the SERCA crystal structure and in the Pma1 homology model. A) Binding pocket of 7 in SERCA. B) The binding pocket of 7 in the Pma1 homology model is larger as compared to the one in the SERCA crystal structure, and it extends deeper into the core of the protein towards transmembrane helix M5 (indicated with a white arrow). Figure E: SERCA•7 •TNPATP crystal structure (pdb: 5NCQ, blue) superposed with the crystal structure of SERCA•Tg •TNPATP crystal structure (pdb: 3AR7, orange). A) Overall view of the two crystal structures superposed on transmembrane helices [file pone.0188620.s001.pdf]

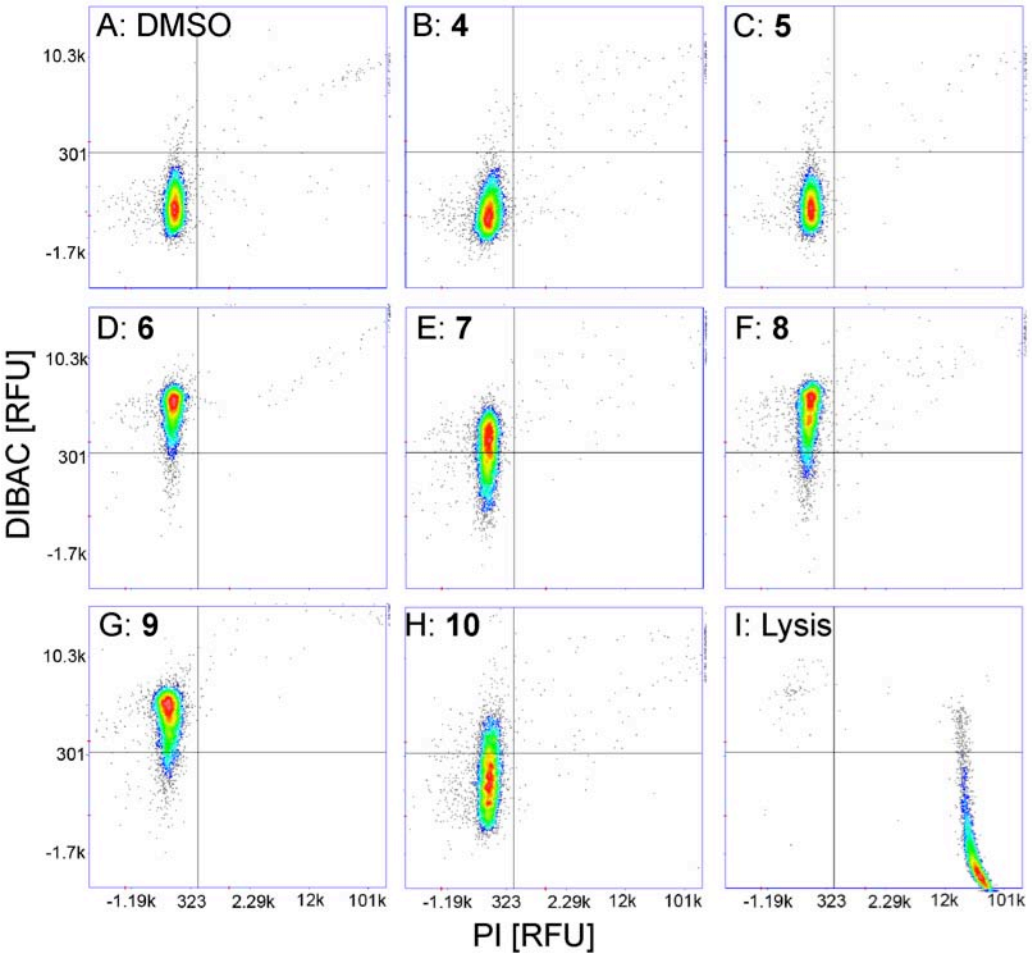

Figure A: Membrane potential and integrity assay.

S. cerevisiae cells incubated for 30 min with A: 1 % DMSO, B-H: 10 μM **4 - 10**, I: Lysis buffer. The 4 quadrants define cells as $DiBAC_4(3)$ positive (+, top quadrants) or negative (-, bottom quadrants) and PI positive (+, right quadrants) and negative (-, left quadrants).

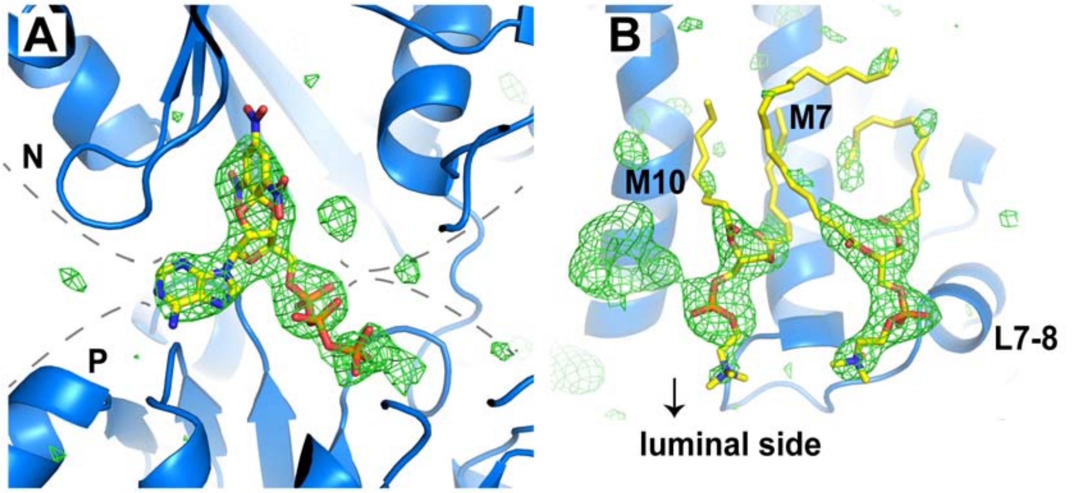

Figure B: Difference electron densities for bound TNPATP and lipids.
A) Bound TNPATP between the N- and P-domain (domain borders indicated by dashed lines). This binding mode is almost identical to previously reports (pdb: 3AR7) (1).
B) Residual electron density indicated the presence of two lipid molecules at the luminal membrane interface. In accordance to the expected abundance in a sample derived from SR membrane, two DOPC molecules were modeled. The green mesh is an *m*Fo-*D*Fc electron density map calculated before the ligands were added to the model, and contoured at 3.0 σ and 2.8 σ for TNPATP and DOPC, respectively.

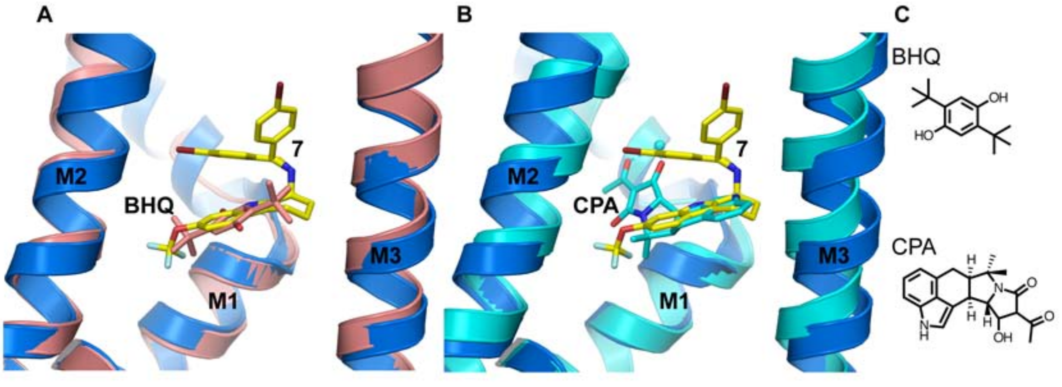

Figure C: Compound 7 binds in the same pocket as BHQ and CPA in SERCA.
Structural alignment of SERCA•7•TNPATP (pdb:5NCQ, blue) with A) SERCA•BHQ (pdb:2AGV, salmon) and B) SERCA•CPA (pdb:3FGO, cyan) (2, 3). The compound is cradled between M1, M2, M3 and M4 (behind the viewpoint) inside the cavity marking the cytoplasmic end of the Ca^{2+} inlet channel of SERCA. The view is from the inside of the cavity, for clarity. C) Chemical structures of BHQ and CPA.

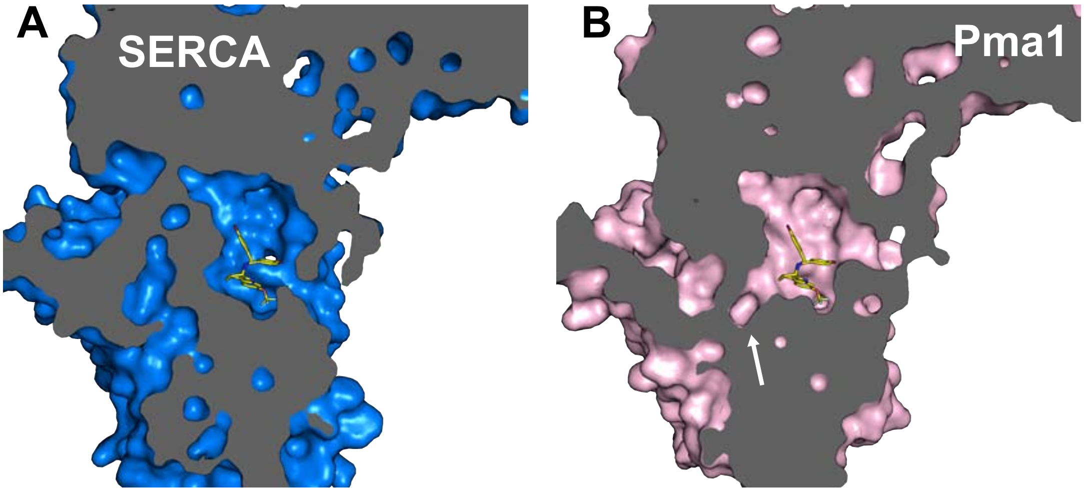

Figure D: Binding pocket of 7 in the SERCA crystal structure and in the Pma1 homology model. A) Binding pocket of **7** in SERCA. B) The binding pocket of **7** in the Pma1 homology model is larger as compared to the one in the SERCA crystal structure, and it extends deeper into the core of the protein towards transmembrane helix M5 (indicated with a white arrow).

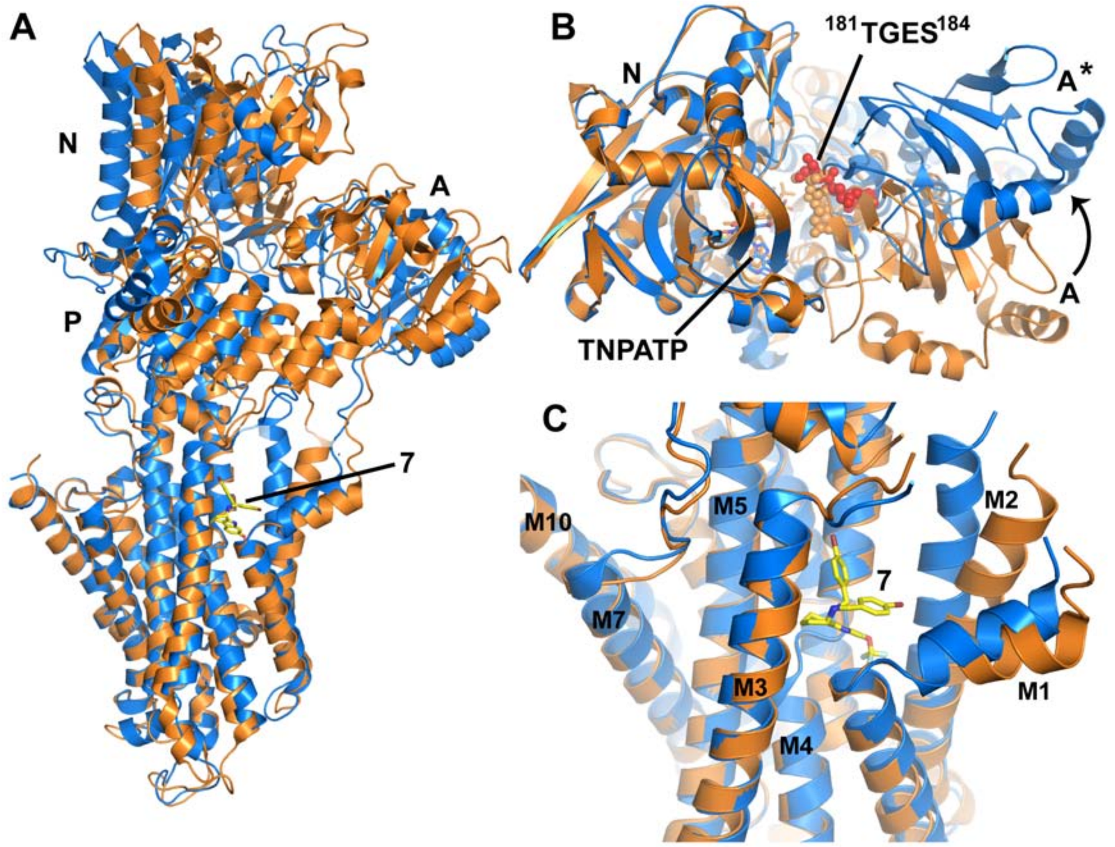

Figure E: SERCA•7 •TNPATP crystal structure (pdb: 5NCQ, blue) superposed with the crystal structure of SERCA•Tg •TNPATP crystal structure (pdb: 3AR7, orange). A) Overall view of the two crystal structures superposed on transmembrane helices 7-10. All three cytosolic domains have undergone a positional shift. B) Top view on the N- and A-domains, superposed on the N-domain, illustrating the considerable displacement of the A domain (labeled A* in the SERCA•7•TNPATP structure), including the functionally important so-called TGES-loop (residues 181-184, red and orange spheres). C) Displacement of transmembrane helices M1 and M2 near the binding site of compound **7**. These displacements translate into a substantial backward movement of the A- domain and smaller displacements of the P- and N-domains as compared to all other known SERCA structures.

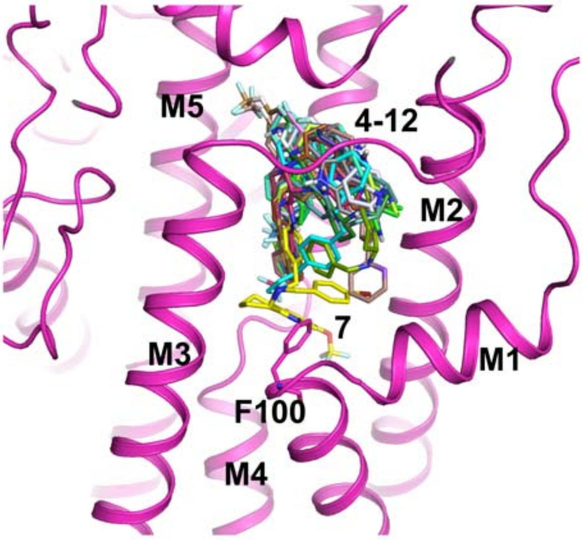

Figure F: Docking of THCs into the Na^+,K^+-ATPase homology model. Structural overlay of compound **7** (yellow) within the SERCA crystal structure with the docking results of compound **4** – **12** docked into the Na^+,K^+-ATPase homology model. None of the compounds gave a docking position similar as to the observed position of **7** in SERCA, due to a steric clash with Phe100, a residue previously discussed to be responsible for the resistance of the Na^+,K^+-ATPase to CPA (Laursen, Bublitz et al, 2009)(3).

V1

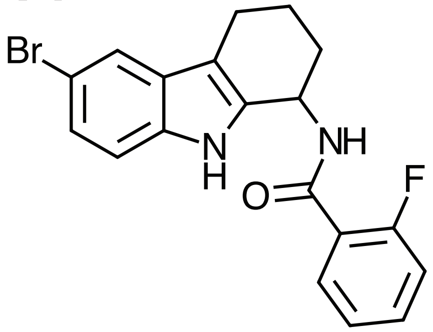

V2

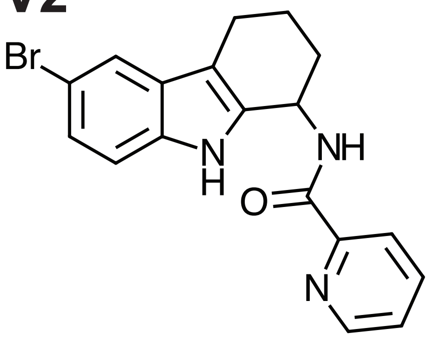

Figure G: Compound V1 and V2 identified as viral inhibitors. Compound 25 and 26 in Gudmundsson et al., 2009 (4).

Table A: ATP hydrolysis and fungal growth effect of V1 and V2.

	ATP hydrolysis IC_{50} [µM]	MIC [µM]	
ID	*S. cerevisiae* Pma1	*S. cerevisiae*	*C. albicans*
V1	>183	>150	>150
V2	>183	>150	>150

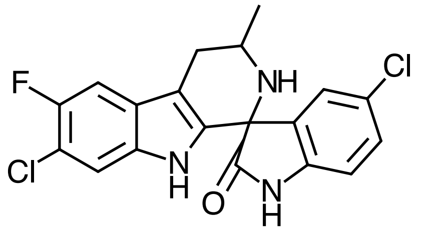

Figure H: The racemic compound of NITD609. Synthesis described in Dandapani et al., 2012 (5).

Table B: ATP hydrolysis and fungal growth effect of the racemic of NITD609.

	ATP hydrolysis IC_{50} [μM]			MIC [μM]		
ID	CaPma1	SERCA	Na^+,K^+-ATPase	*C. albicans*	*C. krusei*	*C. parap-silosis*
Racemate of NITD609	0.15 ±0.02	1.07 ±0.30	46.7 ±7.5	>75	>75	>75

Table C. Data collection and refinement statistics

**Data collection**	
No. of data sets combined	4
Wavelength (Å)	0.90
Space group	$P2_12_12$
Unit cell Dimensions	*a*=125.6 Å<br>*b*=224.6 Å<br>*c*=57.1 Å
Resolution (Å) [a]	56.16 - 3.00 (3.30-3.00)
Unique reflections [a]	61,971 (15,091)
$I/\sigma I$ [a]	15.1 (2.2)
CC (1/2) [a]	99.9 (78.2)
R_{pim} (%) [a]	9.9 (>100)
Completeness (%) [a]	99.4 (97.7)
Redundancy [a]	20.0 (17.1)
**Refinement**	
Resolution (Å)	56.16 – 3.00
No. reflections	61,945
$R_{work}/R_{\mathrm{free}}$ (%)	21.3/26.7
No. atoms	
Protein	7,596
K^+	1
TNP-ATP	46
Compound 7	36
DOPC	84
Water	2
Avrg. B-factors	
N-/P-/A-domain	89.6/74.0/109.7
TM-domain	95.4
K^+	135.5
TNP-ATP	104.4
Compound 7	101.6
DOPC	89.9
Water	67.9
R.m.s. deviations	
Bond lengths (Å)	0.003
Bond angles (°)	0.63

[a] Numbers in parentheses refer to the highest resolution shells as indicated

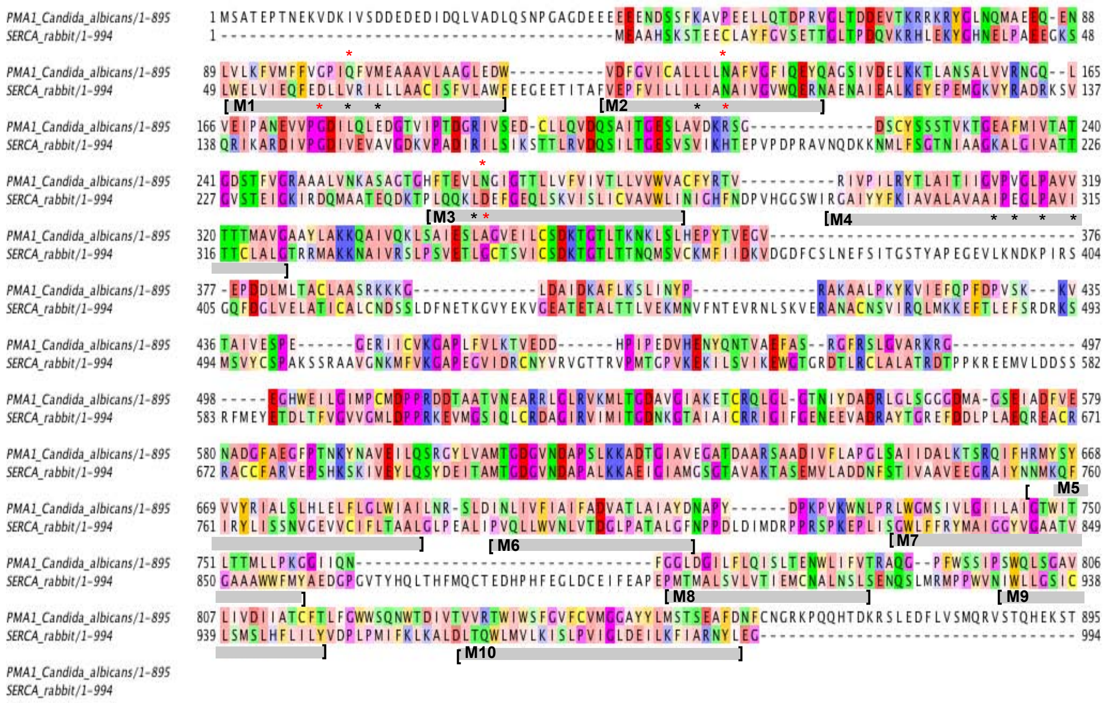

Figure I: Sequence alignment of *Candida albicans* Pma1 and rabbit SERCA used as basis for homology modeling. Residues with polar interactions to compound **7** and their equivalents in the Pma1 homology model are marked with red asterisks, other residues surrounding the binding site are marked with black asterisks. Note that Q101 in the Pma1 model is at an almost equivalent position as SERCA D59.

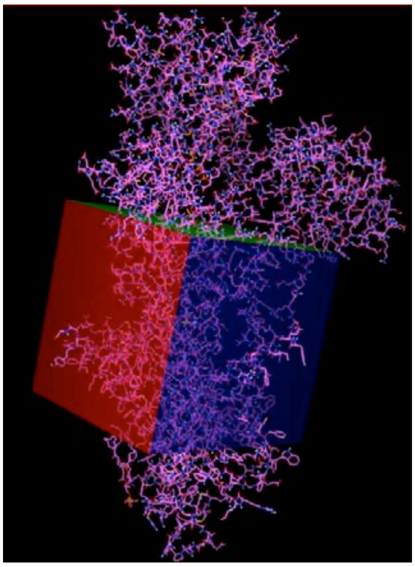

Figure J: Graphical representation of the grid box used as sampling space for docking with AutoDock/Vina.

Chemical synthesis of THCs

3-(Trifluoromethoxy)phenyl]hydrazine hydrochloride (Intermediate)

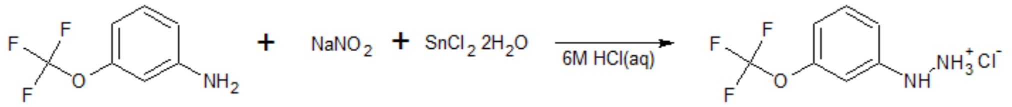

3-(Trifluoromethoxy) aniline (10 g; 56.5 mmol) was dissolved in 6 M hydrochloric acid (120 ml), after which an aqueous solution of sodium nitrite (4.67 g; 67.6 mmol; 10 mL) was added dropwise over 20 min while keeping the temperature at -5°C. To the mixture was added a solution of Tin (II) chloride dehydrate (50g; 225 mmol) in 6 M hydrochloric acid (100 mL) at -5°C. The mixture was stirred at -5°C for another 2 hr, and the resulting precipitate collected by filtration, washed with 0.1 M hydrochloric acid and dried under reduced pressure to afford the title compound (5.93 g; 41.4%).

(2Z)-2-[[3-(trifluoromethoxy)phenyl]hydrazono]cyclohexanone (Intermediate)

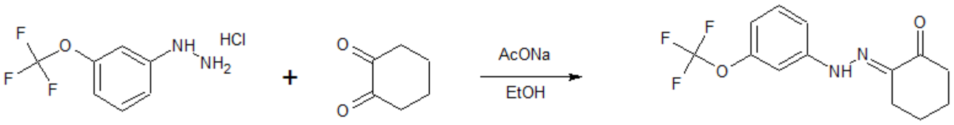

To a solution of cyclohexan-1,2-dione (5.89 g; 52.5 mmol) in ethanol (100 mL) at room temperature was added sodium acetate (6.50 g; 78.7 mmol) followed by dropwise addition of an aqueous solution of [3-(trifluoromethoxy)phenyl]hydrazine hydrochloride (12.0 g; 52.5 mmol; 100 mL). The resulting slurry was stirred at room temperature for 2 hours followed by refluxing for 2 hours. The cooled mixture was evaporated to a half of volume, diluted by water (200 mL) and extracted with methyl-*tert*-butylether (3 x 50 mL). The organic extract were dried over sodium sulfate and evaporated to dryness affording the title compound (10.0 g; 55% purity ≈ 35% yield) as a black solid. The crude product was used in the next step without further purification.

7-(trifluoromethoxy)-2,3,4,9-tetrahydrocarbazol-1-one (Intermediate)

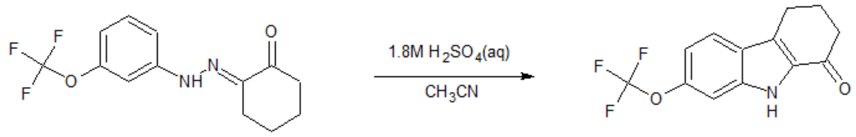

The crude product ((2Z)-2-[[3-(trifluoromethoxy)phenyl]hydrazono]cyclohexanone; 10 g; ≈19.2 mmol) from the previous step was dissolved in acetonitrile (50 mL) and 1.8 M H_2SO_4 (aq) was slowly dropped to it. The reaction mixture was refluxed for 10h, cooled down and poured into cold water (100 mL). The obtained solution was neutralized by adding solid $NaHCO_3$ to pH=5 and was then extracted using EtOAc (3 × 10 mL).
The organic phase was dried over sodium sulfate and evaporated to dryness to yield (4.2g 86% pure; ≈ 70% yield) title product as a black solid used as such in the next step.

Compound 4, 7-(trifluoromethoxy)-2,3,4,9-tetrahydro-1H-carbazol-1-amine hydrochloride

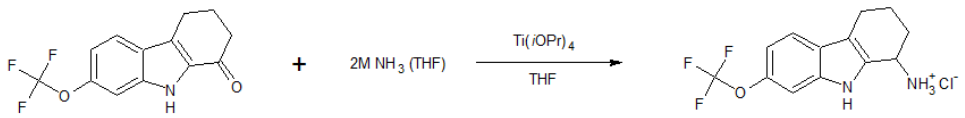

To a solution of 7-(trifluoromethoxy)-2,3,4,9-tetrahydrocarbazol-1-one (2.0 g, 7,43 mmol) in tetrahydrofuran under inert atmosphere (10 mL) was added 2.0 M ammonia in tetrahydrofuran (20 mL) at 0°C. Titanium(IV) isopropoxide (4.22 g, 14,86 mmol) was added inert and the reaction mixture was stirred for 16 hours at room temperature. The reaction mixture was then diluted with ethanol (15 mL), added sodium borohydride (0.85 g, 22,29 mmol) and stirred for 2 hours at room temperature. The reaction mixture was quenched with aqueous. Ammonium hydroxide (20 mL), filtered through a celite pad and the top layer of the pad was washed with ethylacetate (2 x 250 mL). The filtrate was extracted with ethyl acetate, and the combined organic phases was washed with brine solution (100 mL) after which the organic layer was dried over dry sodium sulphate, filtered and evaporated to dryness under reduced pressure to afford a solid of 2.0 g. The resulting solid was acidified with 2.0 M HCl in diethylether. The precipitated solids were isolated by filtration and dried in vacuum to afford an off-white solid 7-(trifluoromethoxy)-2,3,4,9-tetrahydro-1H-carbazol-1-amine hydrochloride (1,6g; 70% yield)
1H NMR (400 MHz,DMSO-d6) δ 11.06(s, 1H), 7.48(d, *J*= 8.8 Hz 1H), 7.34 (s, 1H), 6.95 (d, *J*= 8.8 Hz, 1H), 5.74 (broad, 2H), 4.23 (m, 1H), 2.67 (m, 2H), 2.13-2.02 (m, 2H), 2.00-1.94 (m, 1H), 1.81-1.69 (m, 2H). MS (ESI-*pos*) *m/z* 254.0 $(M+1)^+$ (de-aminated fragment)

Compound 5 *N*-(1-methyl-4-piperidyl)-7-(trifluoromethoxy)-2,3,4,9-tetrahydro-1H-carbazol-1-amine

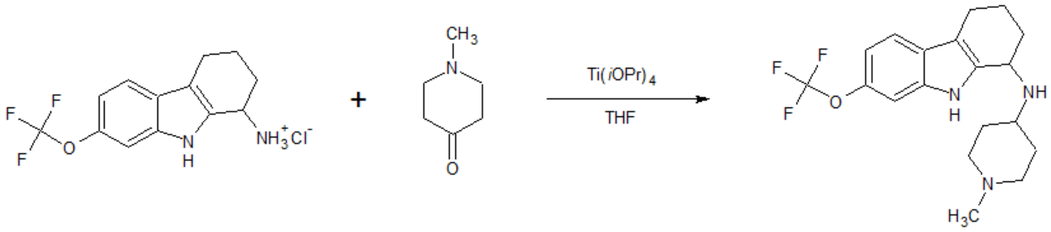

To a solution of 1-methylpiperidin-4-one (100 mg; 0.880mmol) in dry tetrahydrofuran (5 mL) was added titanium(IV) isopropoxide (502.32 mg; 1,77 mmol) at 25 °C. The mixture was stirred for 5 minutes and added solution of 7-(trifluoromethoxy)-2,3,4,9-tetrahydro-1H-carbazol-1-amine hydrochloride (270 mg; 0.88 mmol) and *N*,*N*-diisopropylethylamine (0.46 mL; 2.64 mmol) in dry tetrahydrofuran (5 mL) prepared separately at 25 °C .

After stirring overnight, the reaction mixture was diluted with ethanol (10 mL) and added sodium borohydride (100 mg; 2.64 mmol) at 0°C. The reaction mixture was allowed to reach room temperature and stirred for 2 hours after which the reaction mixture was quenched with saturated aqueous ammonium hydroxide (20 mL) and filtered through a pad of celite. The top layer of the pad was carefully washed using ethylacetate (2 x 250 mL) and the combined filtrates were extracted with ethyl acetate. The organic layer was washed with brine solution (100 mL), dried over anhydrous sodium sulphate, filtered and evaporated to dryness under reduced pressure to give crude product. The crude product was purified by preparative HPLC purification to yield *N*-(1-methyl-4-piperidyl)-7-(trifluoromethoxy)-2,3,4,9-tetrahydro-1H-carbazol-1-amine (60 mg; 0.163mmol, 19% yield)

1H NMR (400 MHz,DMSO-d6) δ 10.80(s, 1H), 7.41(d, *J*= 8.3 Hz 1H), 7.26 (s, 1H), 6.89 (d, *J*= 8.3 Hz, 1H), 3.97 (m, 1H), 2.79 (m, 2H), 2.68 (m, 1H), 2.20 (s, 3H), 2.03-1.90 (m, 6H), 1.77-1.61 (m, 4H), 1.49 (m, 2H). MS(ESI-*neg*) *m/z* 366.1 $(M-1)^-$

Compound 10, *N*-([1,1'-biphenyl]-4-ylmethyl)-6-chloro-2,3,4,9-tetrahydro-1H-carbazol-1-aminehydrochloride

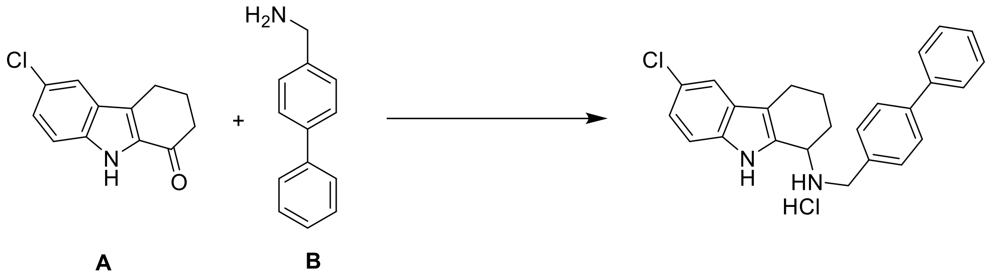

To a solution of A (0.3 g; 1.36 mmol) in THF, titanium tetraisopropoxide (0.83 ml; 2.7 mmol) and compound B (0.38 g, 2.04 mmol) were added and stirred overnight at room temperature. Ethanol (3 mL) was added followed by sodium borohydride (0.2 g; 5.4 mmol) and was stirred for additional 4 hours at room temperature. After completion, reaction was quenched with saturated aqueous NH_3 and stirred for 30 min. The mixture was extracted with EtOAc, filtered through celite and concentrated under reduced pressure. The crude material was purified by column chromatography and converted to HCl salt to yield the title compound (0.05 g; 8.6%).

1H NMR (400 MHz,DMSO-d6) δ 11.54 (s, 1H), 9.78 (brs, 1H), 9.65 (brs, 1H), 7.74-7.68 (m, 6H), 7.56-7.37 (m, 5H), 7.15 (dd, J = 8.8, 2Hz, 1H), 4.68-4.72 (m, 1H), 4.36-4.32 (m, 2H), 2.76-2.62 (m, 2H), 2.33-2.07 (m, 3H), 1.83-1.79 (m, 1H). MS (ESI-*neg*) *m/z* 385.3 $(M-1)^-$.

Compound 9, *N*-(4-(pyridin-2-yl)benzyl)-6-(trifluoromethoxy)-2,3,4,9-tetrahydro-1H-carbazol-1-amine

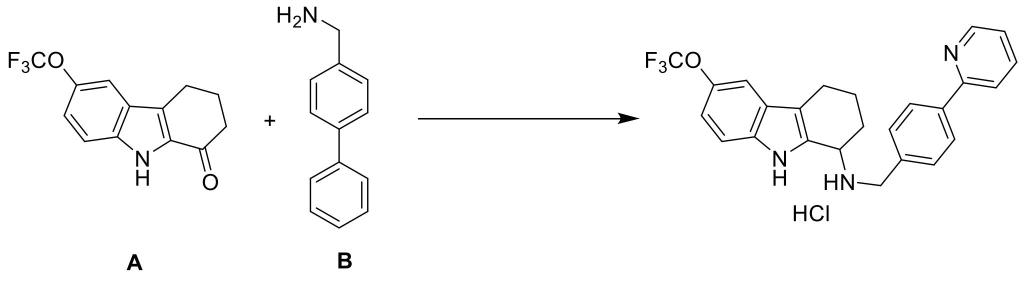

The procedure for Compound **7** was used. The product was converted to HCl salt by trituation as described below.
To 0.4 g (1 mmol) of free base was added MeOH-HCl (15 mL) and stirred at room temperature for 1 hour. The solvent was removed under reduced pressure and the obtained solid was washed with Et_2O (0.4 g; 95%).

1H NMR (400 MHz,DMSO-*d6*) δ 11.82 (brs, 1H), 10.15 (brs, 1H), 10.03 (brs, 1H), 8.81 (s, 1H), 8.32-8.30 (m, 2H), 8.28-8.19 (m, 2H), 7.90-7.88 (m, 2H), 7.78-7.74 (m, 1H), 7.48-7.45 (m, 2H), 7.15-7.10 (m, 1H), 4.75-4.69 (m, 1H), 4.40-4.37 (m, 2H),

2.77-2.75 (m, 2H), 2.40-2.19 (m, 3H), 1.90-1.80 (m, 1H). MS(ESI-*pos*) *m/z* 438.2 $(M+1)^+$.

Compound 6, N-(4-chlorobenzyl)-7-(trifluoromethoxy)-2,3,4,9-tetrahydro-1H-carbazol-1-amine hydrochloride

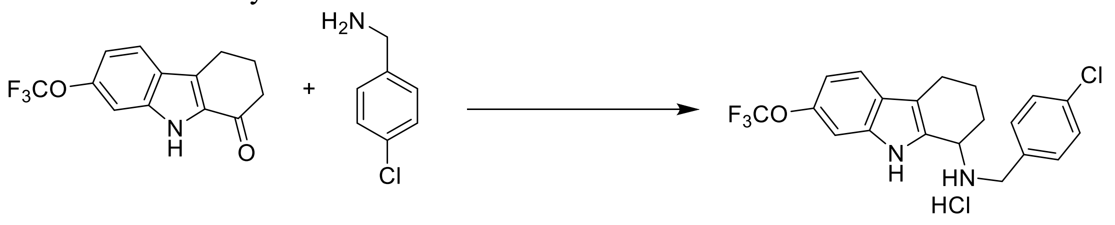

Same procedure as **10**. Product was converted to HCl salt (0.1 g; 50%).

1H NMR (400 MHz,DMSO-*d6*) δ 11.36(s, 1H), 9.50-9.42(brd, 2H), 7.65-7.63(m, 2H), 7.60-7.58(m, 2H); 7.53-7.51(m, 1H), 7.43 (s,1H), 7.02 (d, *J*= 8.9 Hz, 1H), 4.68(br s, 1H), 4.35-4.30 (m, 2H), 2.78-2.66 (m, 2H), 2.21-2.17 (m, 2H), 2.06(m, 1H), 1.86-1.85(m, 1H). MS (ESI-*neg*) *m/z* 393.2 $(M–1)^-$.

Compound 8, *N*-(2-(4-chlorophenoxy)ethyl)-7-(trifluoromethoxy)-2,3,4,9-tetrahydro-1H-carbazol-1-amine hydrochloride

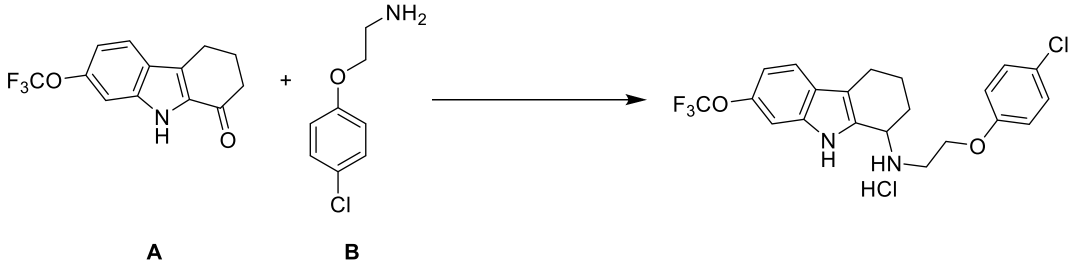

Same procedure as **10**. Product was converted to HCl salt (0.09 g, 23%).

1H NMR (400 MHz,DMSO-*d6*) δ 11.51(s, 1H), 9.73 (br s,1H), 9.60 (br s,1H), 7.59 (d, *J* =8.8 Hz, 1H), 7.41(s, 1H), 7.38-7.35(m, 2H), 7.04-7.00(m, 3H), 4.73 (s,1H), 4.36-4.34 (m, 2H), 3.47 (s, 2H),2.73-2.67 (m, 2H), 2.18-2.17 (m, 2H), 2.09-2.06 (m, 1H),1.84-1.81 (m, 1H).MS (ESI-*neg*)$^-$ *m/z* 423.1 $(M−1)^-$.

Compound 12, N-([1,1'-biphenyl]-4-ylmethyl)-6-chloro-Nmethyl-2,3,4,9-tetrahydro-1H-carbazol-1-amine

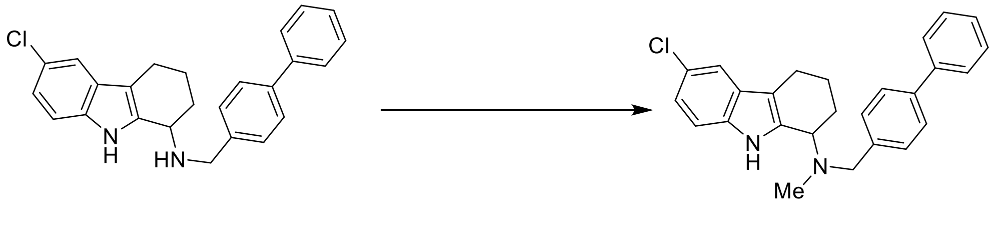

10 (0.1 g; 0.25 mmol) in DMF (10 mL) was added with K_2CO_3 (0.035 g; 0.25 mmol) and MeI (0.073 g; 0.516 mmol) and stirred at room temperature overnight. The reaction mixture was added ice water and extracted with Et_2O. The organic layer was concentrated under reduced pressure and the crude product was purified by column chromatography (0.020 g; 19%).

1H NMR (400 MHz,DMSO-d6) δ 10.88(s, 1H), 7.67-7.57(m, 6H), 7.47-7.33(m, 5H), 7.01 (dd, *J*= 8.4, 2.0 Hz, 1H),4.18-4.12 (m, 1H), 3.65 (s, 2H), 2.67-2.56 (m, 2H), 2.13(s, 3H), 2.03-1.98(m, 2H), 1.83-1.80(m, 1H), 1.67-1.62(m, 1H). MS (ESI-*neg*) *m/z* 399.1 $(M-1)^-$.

Compound 7, N-(4-bromobenzyl)-9-methyl-7-(trifluoromethoxy)-2,3,4,9-tetrahydro-1H-carbazol-1-amine hydrochloride)

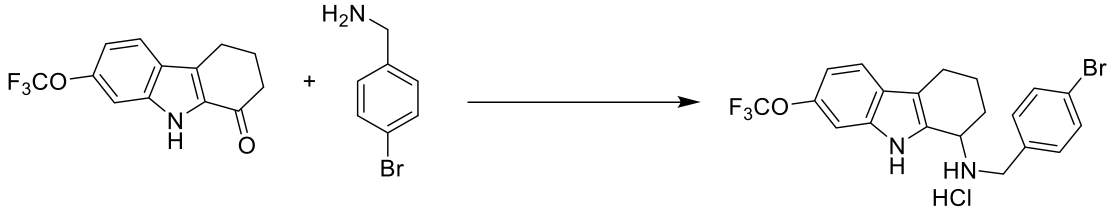

A **B**

Same procedure as **10**. Product was converted to HCl salt (0.1g, 38%).

1H NMR (400 MHz,DMSO-d6) δ 11.47 (s, 1H), 9.65 (s, 1H), 9.55 (s, 1H), 7.66 (d, J = 8.8 Hz, 2H), 7.60-7.58 (m, 3H), 7.42 (s, 1H), 7.02 (d, J = 8.8 Hz, 1H), 4.69 (s, 1H), 4.30 (s, 2H), 2.77-2.65 (m, 2H), 2.33-2.20 (m, 2H), 2.18-2.05 (m, 1H), 1.85-1.81 (m, 1H). MS (ESI)*m/z* 437.2 (M^+-2); HPLC (PDA 235.0 nm), 97.13%

Compound 11, N-([1,1'-biphenyl]-4-ylmethyl)-6-chloro-9-methyl-2,3,4,9-tetrahydro-1H-carbazol-1-amine hydrochloride)

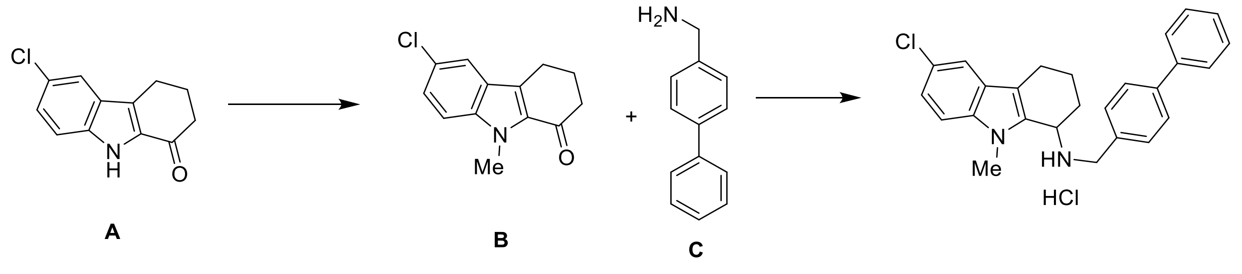

Synthesis of intermediate: To a solution of 6-chloro-2,3,4,9-tetrahydro-1H-carbazol-1-one (A) (1g, 4.55 mmol) in THF (25 mL) was added cesium carbonate (3g, 9.1 mmol) and the slurry was stirred at 0 °C for 5 minutes. Methyl iodide (0.78g, 5.46 mmol) was added and stirred at rt for 3h. The reaction mixture was diluted with ethyl acetate, washed with water and then with brine and dried over sodium sulfate. The organic layer was removed under reduced pressure to afford the desired product (0.7g, 66%).

The title compound was synthesized using same procedure as **10**. The product was converted to HCl salt (0.1 g, 19.4%).

1H NMR (400 MHz,DMSO-d6) δ 7.67-7.62 (m, 4H), 7.52-7.32 (m, 7H), 7.08 (dd, J = 8.8, 2.0 Hz, 1H), 3.97-3.82 (m, 3H), 3.67 (s, 3H), 2.72-2.68 (m, 1H), 2.50-2.42 (m, 1H), 2.34 (bs, 1H), 2.23-2.20 (m, 1H), 1.97-1.93 (m, 1H), 1.77-1.62 (m, 2H); MS (ESI)*m/z* 401.2 $(M+1)^+$

1. Toyoshima, C., Yonekura, S.-I., Tsueda, J., and Iwasawa, S. (2011) Trinitrophenyl derivatives bind differently from parent adenine nucleotides to Ca2+-ATPase in the absence of Ca2+. *Proc. Natl. Acad. Sci. U. S. A.* **108**, 1833–1838
2. Obara, K., Miyashita, N., Xu, C., Toyoshima, I., Sugita, Y., Inesi, G., and Toyoshima, C. (2005) Structural role of countertransport revealed in Ca(2+) pump crystal structure in the absence of Ca(2+). *Proc. Natl. Acad. Sci. U. S. A.* **102**, 14489–14496
3. Laursen, M., Bublitz, M., Moncoq, K., Olesen, C., Møller, J. V., Young, H. S., Nissen, P., and Morth, J. P. (2009) Cyclopiazonic acid is complexed to a divalent metal ion when bound to the sarcoplasmic reticulum Ca2+-ATPase. *J. Biol. Chem.* **284**, 13513–13518
4. Gudmundsson, K. S., Sebahar, P. R., Richardson, L. D., Catalano, J. G., Boggs, S. D., Spaltenstein, A., Sethna, P. B., Brown, K. W., Harvey, R., and Romines, K. R. (2009) Substituted tetrahydrocarbazoles with potent activity against human papillomaviruses. *Bioorg. Med. Chem. Lett.* **19**, 3489–3492
5. Dandapani, S., Comer, E., Duvall, J. R., and Munoz, B. (2012) Hits, leads and drugs against malaria through diversity-oriented synthesis. *Future Med. Chem.* **4**, 2279–2294
